# Online GPC monitoring for batch and flow polymerisation reactions†

**DOI:** 10.1039/d5py00554j

**Published:** 2025-06-30

**Authors:** William Pointer, Rowan Radmall, Owen Tooley, James Town, Dennis C. J. Haggart, Zhichun Zhai, Xiaofan Yang, Daniel W. Lester, Paul Wilson, David M. Haddleton

**Affiliations:** a Department of Chemistry, University of Warwick Coventry CV4 7AL UK d.m.haddleton@warwick.ac.uk; b Polymer Characterization RTP, University of Warwick Coventry CV47AL UK

## Abstract

Gel Permeation Chromatography (GPC) is well established as the gold standard for routine molecular weight analysis of polymers giving both mass and mass dispersity information. Online-GPC has the potential to be especially effective when used to monitor the progression of reactions conducted in both batch and flow, within a single synthetic/analysis platform. However, use of this technique has often been limited to custom built systems which can be difficult to reproduce and to use. In this work we present a guide to easily modifying commercially available high pressure liquid chromatography (HPLC)/GPC equipment, to facilitate their application as flow chemistry platforms with integrated online chromatography using commercial software for both instrument control and automatic analysis of multiple GPC traces. We demonstrate this approach as an entry point to conducting simple polymerization techniques using both batch and flow reactions with incorporated automated online monitoring. The work outlined should enable wider adoption of techniques such as online-GPC for real-time monitoring of polymerization reactions which in turn leads to real time data available for both reaction process and product control.

## Abstract 2

Gel Permeation Chromatography (GPC) is well established as the gold standard for routine molecular weight analysis of polymers giving both mass and mass dispersity information.

## Graphical abstract

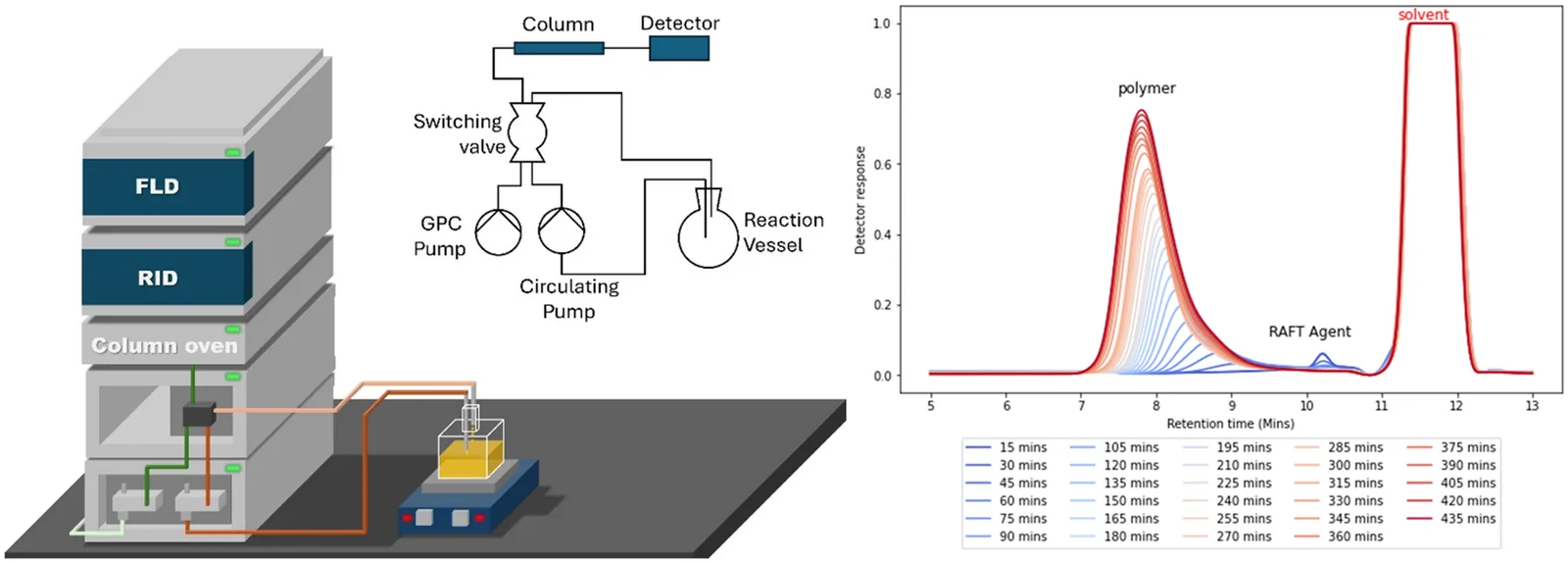

## Introduction

Accurate and reliable determination of the polymer molecular weight and molecular weight distribution is a key step in characterizing the product from a polymerisation reaction. Whilst techniques such as nuclear magnetic resonance (NMR)^1–3^ are capable of producing molecular mass values from diffusion ordered spectroscopy (DOSY) measurements, for example, and other techniques such as viscometry, mass spectrometry, static light scattering (SLS), *etc*. provide accurate molecular mass values, these techniques often offer lower versatility and accessibility and do not always yield values for mass dispersity.^4^ In their recent work Liarou *et al.* have shown how in certain instances both mass and *Đ* values can be attained through advanced electron microscopy, however, it is noted that this technique does not readily lend itself to in-flow analysis.^5^

Thus, GPC remains the routine technique for polymer mass and dispersity determination of choice in most polymer synthesis laboratories and quite often as a quality control measure in commercial polymerisation. GPC can be compatible with most solvents and can be performed at ambient and elevated temperatures using appropriate columns and calibrants suited to most modern applications and polymer types. Minimal sample preparation is required, and relatively reliable results can be achieved through well-calibrated and well-maintained instruments. There are relatively fast acquisition times typically between 15 and 45 minutes allowing for acceptable high sample throughput depending on the number and nature of the columns chosen for separation.

Whilst commonplace as an offline tool, there is a lack of availability and use of GPC for online reaction monitoring. Junkers,^6–8^ Warren^9–11^ and others^12^ have previously demonstrated the concept of online GPC,^13^ however, in these instances their experimental set up frequently makes use of heavily modified, customized or legacy equipment that is often not trivial to replicate and is often built upon customized, but open source, software. Additionally, with few, if any, commercial solutions available, the widespread adoption of online GPC has been minimal.

The use of flow chemistry in polymer synthesis is not that well studied despite many commercial polymerizations being conducted in flow and it is seldom seen in undergraduate courses.^14–16^ In recent years there has been an increasing amount of work demonstrating capabilities of flow chemistry for polymer synthesis showing high throughput screening,^12^ kinetic studies^17^ and the foundations of chemical machine learning,^9,18^ spanning many polymerization techniques.^19–23^

Flow chemistry presents several barriers of entry to researchers unfamiliar with the field who are usually more used to carrying out reactions in batch mode in round bottom flasks or Schlenk tubes, *etc*. “Specialized” flow equipment, while effective, can come with a significant capital cost to entry and potentially lack key features, such as the ability to pre-program experimental conditions or queue a series of experiments. All-in-one solutions offered by some commercial suppliers can work well for established chemical processes, typically with simple maintenance and cleaning procedures, but can function poorly when required to perform procedures more complex than simple steady-state reactions.

More basic flow solutions, such as standalone syringe pumps connected to tubing have been used and have previously shown good efficacy in dosing precise volumes of reagents.^24–26^ However, syringe pumps can swiftly encounter limitations when conducting polymerizations, often due to exotherms, increased pressure or the increase in viscosity of reaction solutions especially at higher concentrations as molecular weight increases. This is due to the relatively poor ability of syringe pumps to dispense solution against a back pressure.^27^ Larger and more complex flow equipment is available commercially but these are often aimed at pilot-scale laboratories and come at a significant cost and infrastructure requirements.

The use of customized platforms can offer significant advantages, allowing for high degrees of specialization, precise control over commercial devices and often with relatively low start-up costs. However, this approach limits the widescale adoption of flow chemistry in polymer science and requires significant specialized knowledge of computer programming and device control to construct workable systems suitable for complete automation. High pressure liquid chromatography (HPLC) is ubiquitous in chemistry research facilities, with a high proportion of researchers having access to and experience of using such devices. These devices produce high pressure flow with highly accurate and reliable flow rates, and the ability to efficiently mix two or more reaction streams at high pressure and are thus ideal for facilitating flow chemistry reactions.

## Online GPC using “off-the-shelf” HPLC hardware

Online chromatography requires four core devices: (1) A dual-piston HPLC pump, (2) a sampling valve, (3) a separation column and (4) a detector. In this work, “Agilent 1260 infinity 2” HPLC components with modifications carried out in the manner described below were used. This approach should be possible on any HPLC system from any manufacturer, where a 6-port, 2-position switching valve in the auto sampler is accessible, and the required detectors and analysis software are available.

**Fig. 1:**
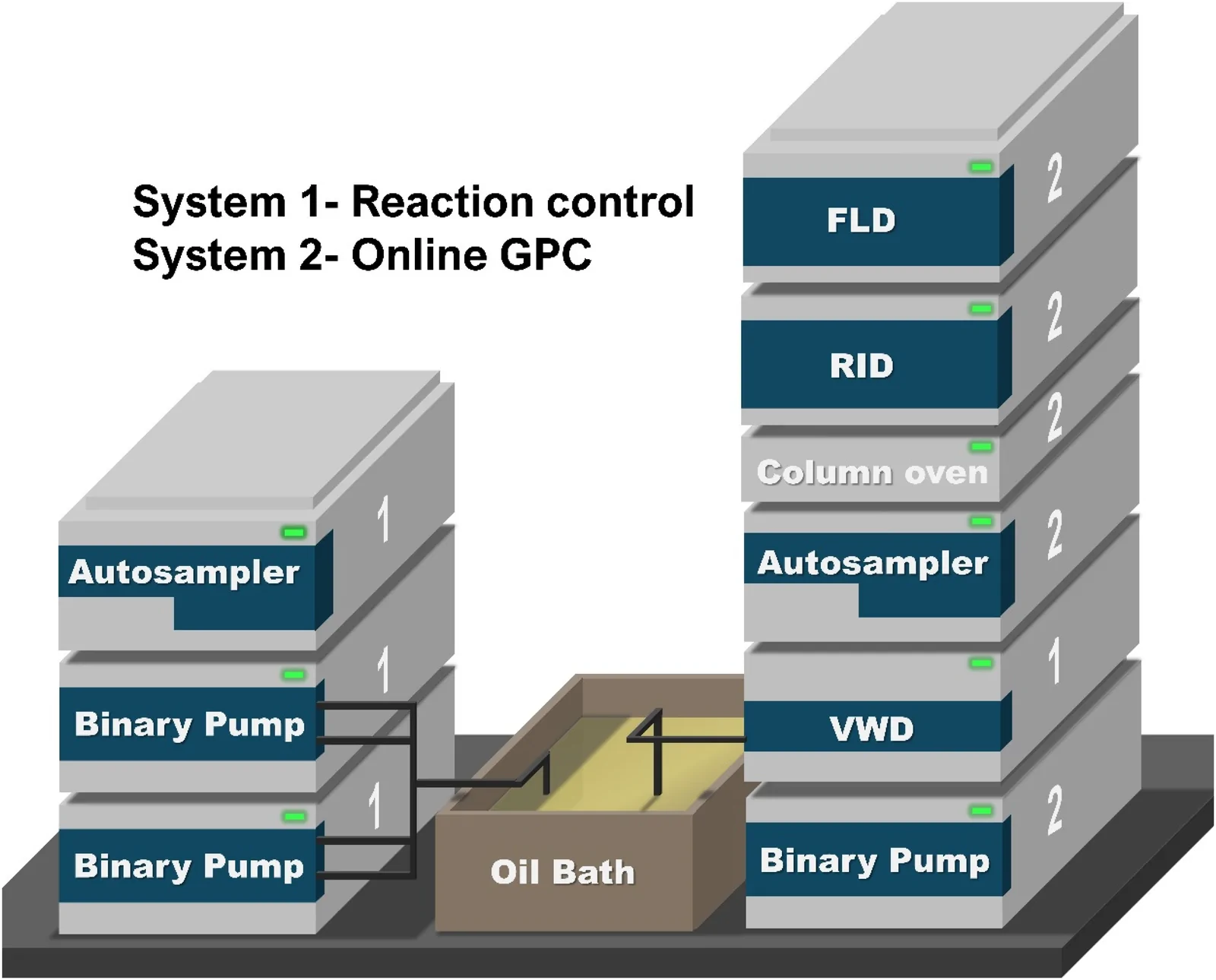
Schematic view of the flow reactor coupled to the online GPC. System 1 is a self-contained HPLC system consisting of two binary pumps, an autosampler and a UV detector. System 2 forms the online GPC, consisting of a binary HPLC pump, an autosampler, column oven fitted with variable wavelength UV (VWD), fluorescence (FLD) and differential refractive index (DRI) detectors.

**Fig. 2:**
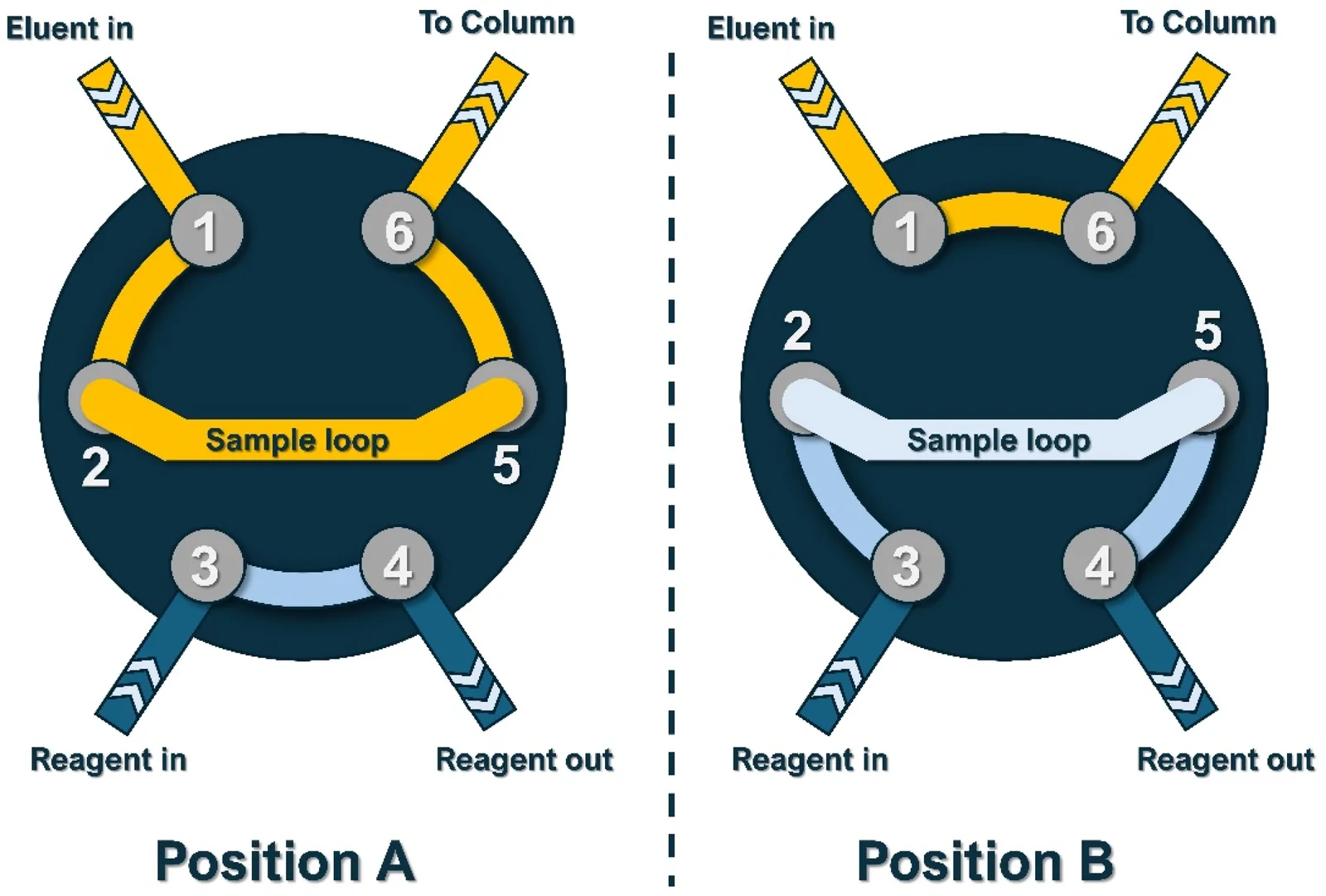
6-Port, 2-position valve configuration for the online GPC/SEC.

Additional modules, such as column ovens and secondary detectors, were employed in the same configurations as their offline equivalents, Fig. 1. The autosampler, used for sample injection into the online GPC, was the only module requiring any intrinsic modification, that being the re-configuration of the 6-port, 2-position switching valve to the configuration described in Fig. 2. During regular operation of the device, the eluent flow is carried by the pump to port-1, through the valve to port-2, the flow is subsequently channeled to port-5 through a predefined volume sample coil and finally returns through the valve to port-6 where it proceeds to the column. Port-3 hosts the sample inlet line, with port-4 the sample outlet line.

Within this configuration sample injection is achieved *via* two switches of the valve: the first switch, position A to B, the eluent flow from port-1 to port-6. While the sample inlet is passed from port-3 to port-2, through the sample coil to port-5, then out from port-4. The volume of sample within the sample coil is then injected on to the second switch and then returned to position A. The actual volume of sample present within the sample coil is determined by the flow rate of the sample, the volume of the sample coil and the delay between valve switches. In this work a fixed delay of 90 seconds was used, and the volume of the sample coil was chosen according to the lowest flow rate used during an experiment.

The pumps, column ovens and detectors were not modified, allowing the use of Agilent *OpenLabs* software to control and run the GPC/SEC hardware and to carry out the data analysis, whilst simultaneously running experimental methods on a second instance of the software.

Agilent OpenLabs software further allows for centralized control over a significant amount of both modern and legacy equipment, such that the operator can rapidly modify the experimental set up without the need for advanced coding knowledge using the in-built sub routines. Experiments were conducted in OpenLabs by creating “*methods*” for each set of experimental conditions and for each set of analytical procedures, with each method set being run in sequence. It is important to note that interestingly OpenLabs absolutely requires the physical presence of autosampler, pump and detector modules in order to run a method, whether or not those devices are used in the experiment/analysis.

The flexibility of OpenLabs and the trivial hardware modification allows for the return of the device to offline analysis modes without requiring extensive work, thus facilitating regular re-calibration, maintenance and use of the device to monitor batch processes in real time as well as flow processes. This also means that researchers who want to gain the benefits of online monitoring of flow analysis but who may lack the budget or prior experience can gain access to the techniques with little financial or capital risk. It is noted that all HPLC systems that are modified in this manner are not “*locked into*” this role and can rapidly be returned to use as traditional HPLC/GPC systems.

The appropriate choice of pump is essential for efficiently conducting reactions in flow and whilst not the truly optimal solution, the dual piston pumps found in most HPLC/LC systems provide a low pulsation and steady flow profile over a large range of flow rates and are usually highly chemically compatible, usually making them an excellent choice for flow chemistry. In this work all pumps used were Agilent binary pumps, fitted with sapphire pistons and PTFE piston seals. The choice of binary pumps (two pump units per device) *vs.* isocratic or quaternary pumps was to enable the highest level of control over reagent and eluent streams for the lowest monetary cost.

## GPC detectors, column selection and calibration

GPC, reduced to its simplest form, requires a stable isocratic pump, a switching valve, a column, and a detector. There are a range of detectors commonly used for GPC, refractive index detection (RID) is by far the most common with viscometers, UV and light scattering also being prevalent in offline analysis.

Arguably the simplest method for conducting GPC is to use a single RID following the column(s) using a calibration curve that relates retention time/volume eluted to mass derived from a set of narrow disperse polymers used as calibration standards of appropriate polymers sourced from a range of suppliers. Column selection in GPC is non-trivial and can strongly influence the quality of the data produced. In offline GPC, typically either a single or double column arrangement is employed in series, along with a “*guard*” column which can help to extend the life of the chromatographic columns by protecting the analytical columns from any material which may damage the stationary phase or impede the flow. Analysis is usually carried out at a pumping rate of 1 mL min^−1^ which then equates time in minutes to elution volume. Many different column set ups are commercially available and are typically categorized into either general use columns or high throughput columns. For use in online GPC, a balance must be struck between sampling rate and column resolution.

**Fig. 3:**
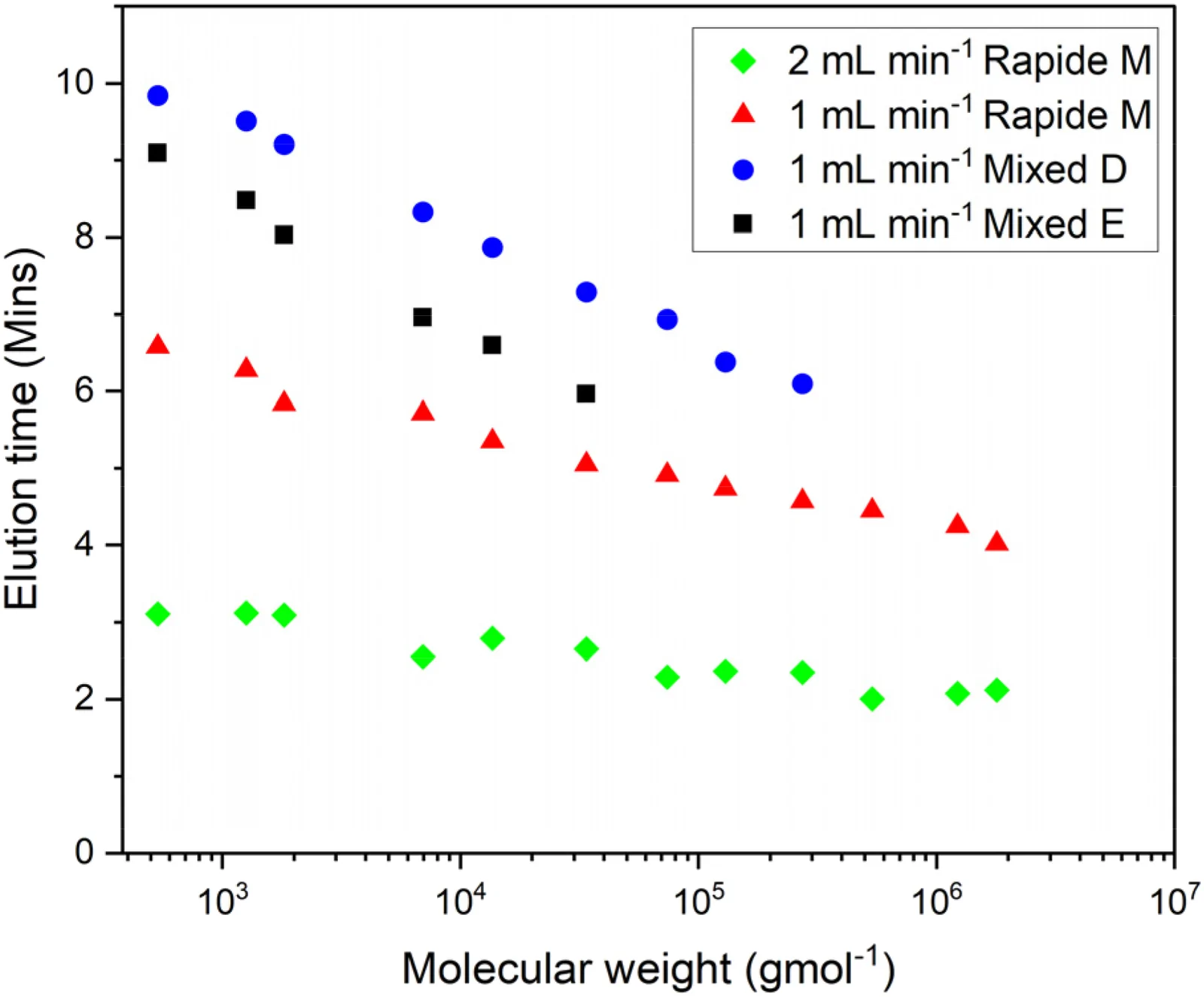
Overlayed calibration samples using a Rapide M at 1 and 2 mL min and Agilent mixed E and D columns at a pumping speed = 1 mL min^−1^.

High throughput columns, such as the Agilent “*Rapide*” have been especially formulated for rapid GPC analysis and have frequently been used in online monitoring solutions. These are designed to operate under high pressure with high flow rates, providing useful for polymerization reactions with high rates of reaction, or in cases where high temporal resolution, every 3–10 minutes, is required (Fig. 3). A potential downside of these columns is often a reduced resolution due to the presence of fewer theoretical plates. This hinders the accuracy of the analytical technique and thus limits its validity for use in producing large data sets.

The inverse of these are the “*general purpose*” columns commonly used in offline analysis. These columns are formulated to provide a range of separating powers with differing mass elution cut offs. Columns can be selected to provide separation in ranges 500–50 000 g mol^−1^ to 500–40 000 000 g mol^−1^ for example. The rate of data acquisition is lower using a “*general purpose*” column compared to a high throughput column, however, the benefit from this is a dramatic increase in the theoretical plates and hence the accuracy of the data.

This can be demonstrated when the calibration sets from each column are compared (Fig. 3). This illustrates the trade-off between temporal resolution and mass resolution through the value of the gradient of the fit derived from the calibration standards. The Rapide calibrations show a shallower gradient than those from the mixed D and E columns, indicating a poorer ability to separate polymers of similar masses.

Ultimately, the choice of these columns comes down to the specific reaction being undertaken, the largest mass likely to be produced and personal preference of the operator. In our experience a single mixed E or D column fitted with an appropriate guard column gave excellent separation with a monitoring interval of 15 minutes which we considered satisfactory for a wide variety of applications (Fig. 3). Unless otherwise stated this work was conducted using a single Agilent PLgel 5 μm Mixed D column providing an adequate resolution range, between 500 and 400 000 Daltons, whilst keeping the individual GPC method <15 minutes. The online GPC/SEC was calibrated using a conventional PMMA EasiVial RWB calibration set.

A further aspect worth noting is sample filtration prior to GPC injection. While it is common practice to filter GPC samples to remove particles that could potentially cause column damage we decided against adding an inline filter. There is no intrinsic need to filter samples, provided that all reagents are completely dissolved in the reaction mixture and that no insoluble material is present that could block the pores of the GPC column in any case it would not be possible for particulates to pass through the columns and reach the detector to influence the data. We chose reactions that we thought would reduce any precipitation but note that an inline filter could be fitted between the sample injection port and the guard column if it was considered important.

## Flow reactors and residence time distributions

Currently, when carrying out flow chemistry, a large array of different flow reactors are available, such as continuous stirred tank reactors (CSTRs), packed-bed reactors, simple tubular reactors, complex static and active mixing tubular reactors and chip reactors.^28^ The choice of reactor exerts a significant influence on the reaction and in turn the reaction can dictate the reactor that should be used, as thermal and mass transport within the reactor can be greatly affected by the internal geometry of the reactor. Simple tubular reactors are the most commonly used, as they are easy to produce, inexpensive and prove efficient for many thermal and photochemically induced reactions.

Tubular reactors can be produced simply by cutting a tube of known internal diameter to a specific length, yielding a desired internal volume and subsequently attaching appropriate connectors to each end to fit into the reagent flow. Alternatively, pre-cut and finished tubing is commercially available in many different materials, shapes and forms. Commercially available tubular reactors often come in the form of volume-defined sample loops which are often designed for both analytical and preparative HPLC and can usually be acquired inexpensively at most required volumes and thus retention times/reaction times.

Material selection is of importance in considering reactions; polytetrafluoroethylene (PTFE), perfluoroalkoxy alkane (PFA) and stainless steel are the most widely available materials options for the wetted components such as reactors and fluid lines. Stainless steel is very versatile, having the highest internal pressure capacity and further being impermeable to both oxygen and moisture and able to withstand high pressures. PFA can be a less expensive alternative to stainless steel, coming at the cost of lower pressure capacity but this can allow for unwanted O_2_ permeation into the flowing reaction solution. Engineering polymers such as PEEK are also available, indeed any material that is resistant to the solvents and reagents used and is impermeable to ingress of water and air can be used if required.

In all flow reactions it is essential to characterize and report the residence time distribution (RTD) of the reactor at the flow rate used in the experiment. This allows for replication of the reaction conditions and understanding of the distribution of reaction times within the steady state conditions. Acquiring an RTD is best achieved in the form of a pulsed injection, and can even be accomplished during an experimental procedure.^29^ Using modified HPLCs, the pulse injection is accomplished by establishing a steady-state flow regime, injecting a tracer into the reactor, and measuring the output using a detector.

**Fig. 4:**
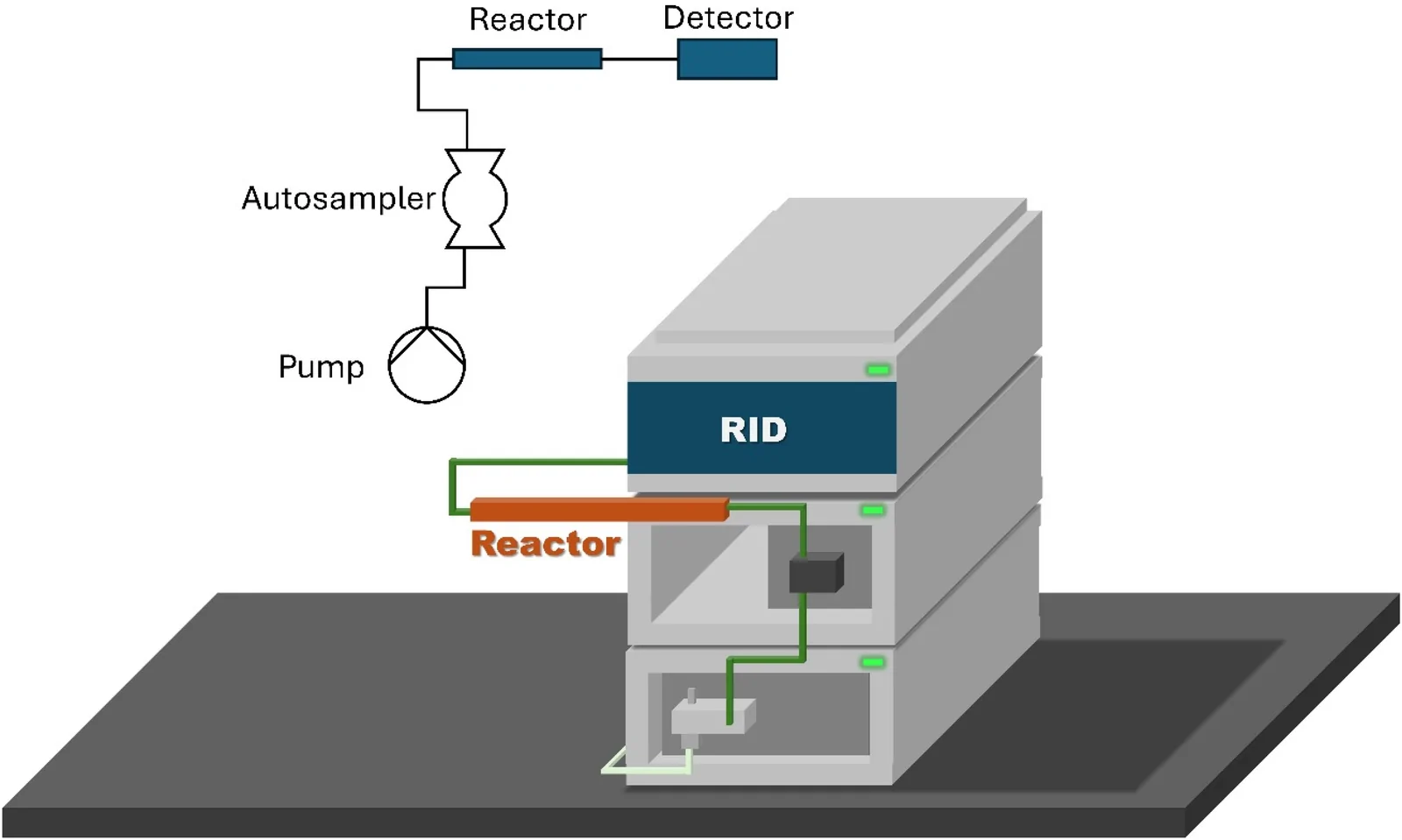
Experimental set up for conducting an RTD using HPLC equipment.

**Fig. 5:**
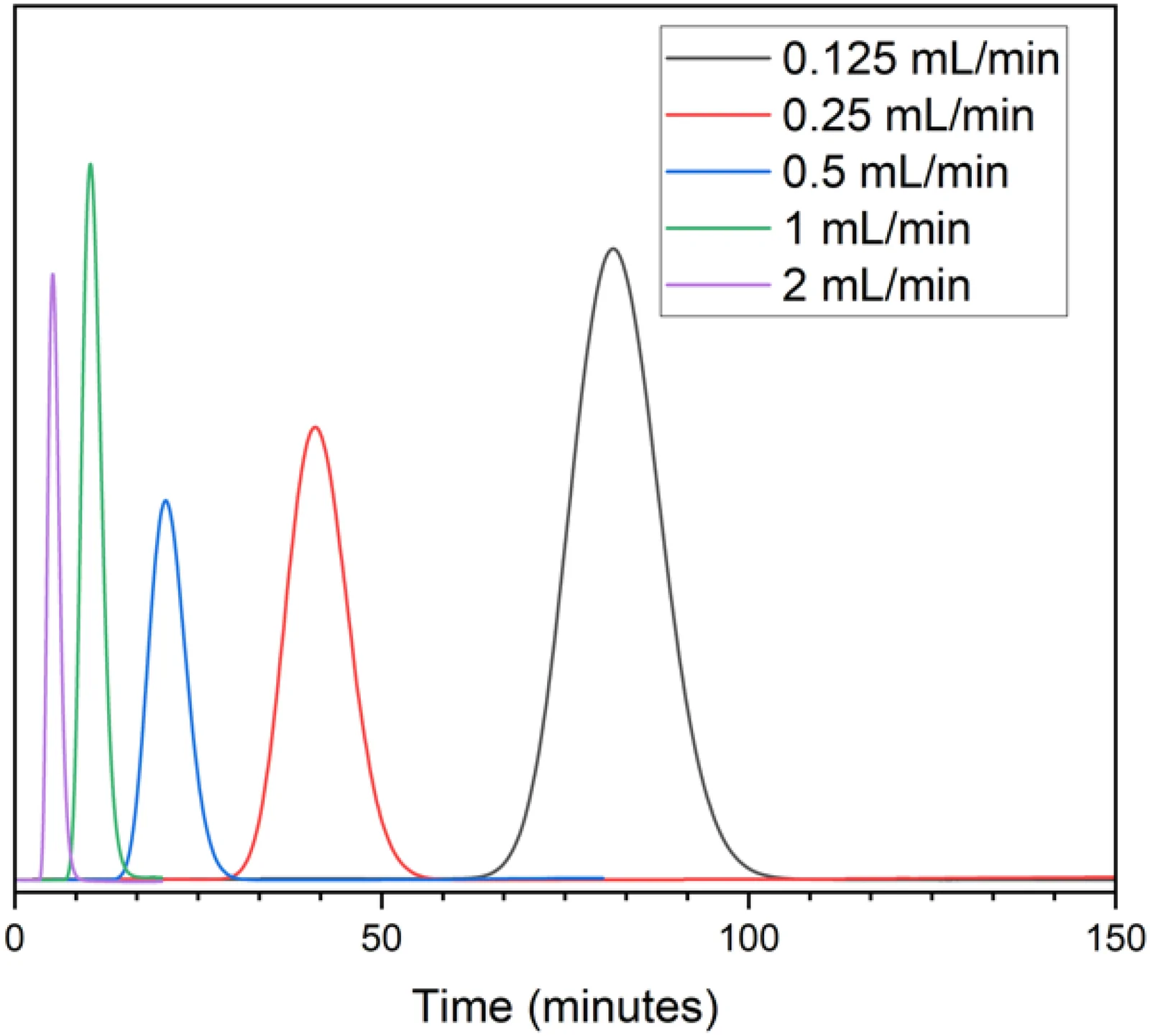
Raw RTD traces of toluene in a 10 mL reactor at flow rates 2 mL min^−1^ to 0.125 mL min^−1^.

To obtain the RTD for the reactors used in this work, a single dual piston pump, autosampler, and RID detector were employed (Fig. 4). RTDs were collected for the specific conditions used for each reactor, with the carrier solvent being pumped at the desired reaction flow rate. For each flow rate a sample of 100 μL of toluene was injected using the autosampler and inserted into the flow line. For conducting RTDs the autosampler was used in its original configuration, and the sample injected from a vial. For the 10 mL reactor used herein, a set of RTDs were measured using a range of flow rates from 0.125 mL min^−1^ to 2 mL min^−1^ (Fig. 5). As would be expected, the average residence time is equal to the volume of the reactor divided by the flow rate. Deviation from this simple formula is also useful in showing problems from poorly calibrated pumps or from poorly maintained reactor units.

**Fig. 6:**
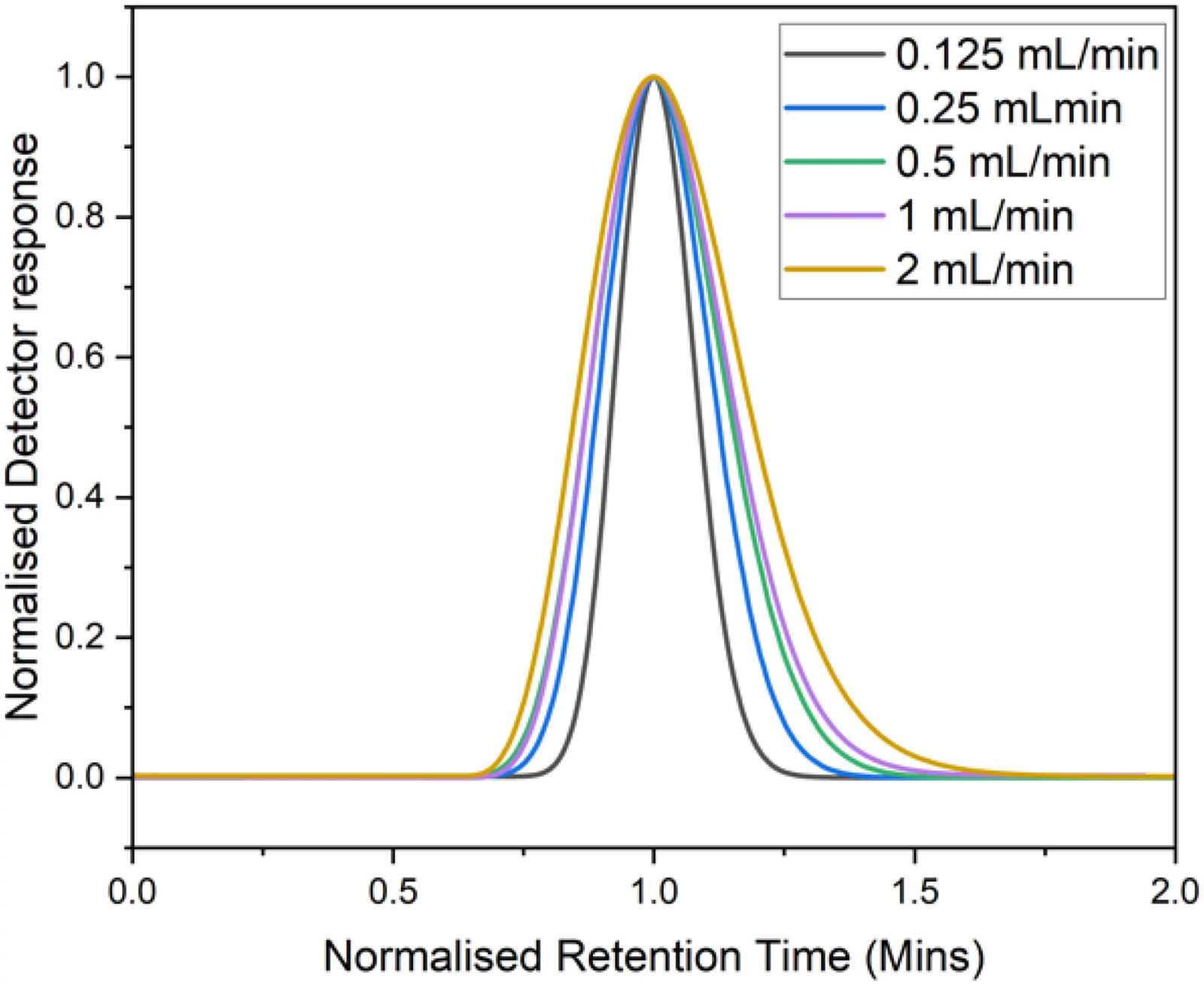
Normalized RTD traces of toluene in a 10 mL reactor at flow rates 2 mL min^−1^ to 0.125 mL min ^−1^.

The RTDs show symmetrical Gaussian peaks caused by the tracer traveling through the reactor and passing into the detector volume (Fig. 5). These RTD traces can be normalized to the average residence time to enable comparisons between the width of the distributions (Fig. 6). In this case the distribution widened at larger flow rates, likely due to a more pronounced parabolic flow profile.

A larger relative deviation at higher flow rates is generally expected, however, the detector reading can often appear to show the inverse, due to the higher flow rate causing the time taken for the distribution to elute to be lower and thus produce a lower absolute deviation. From these RTDs valuable insights can be obtained such as the average residence time and Péclet number (*P*_e_, eqn (3)). The *P*_e_ is a measure of the mixing within a flowing media, describing the relationship between advective transport (through the tube) and the diffusive transport (axial movement across the tube), Fig. 7.1
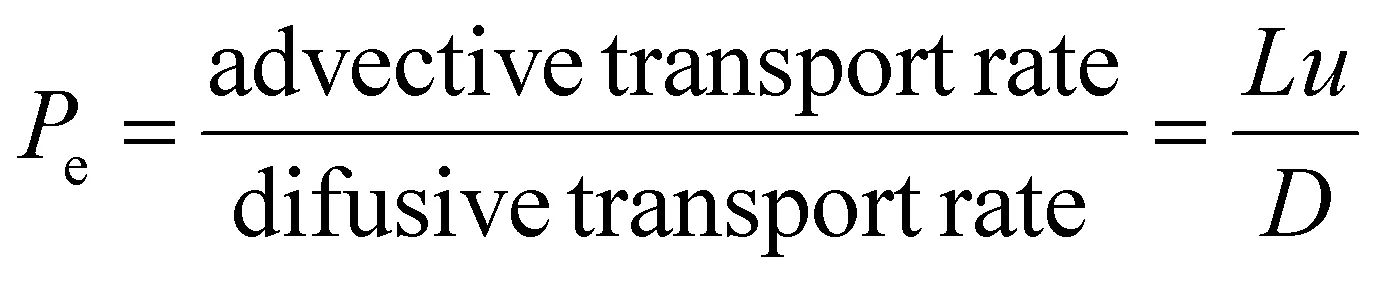
where *P*_e_ = Peclet number, *L* = reactor characteristic length, *u* = local flow velocity and *D* = mass diffusion coefficient.

**Fig. 7:**
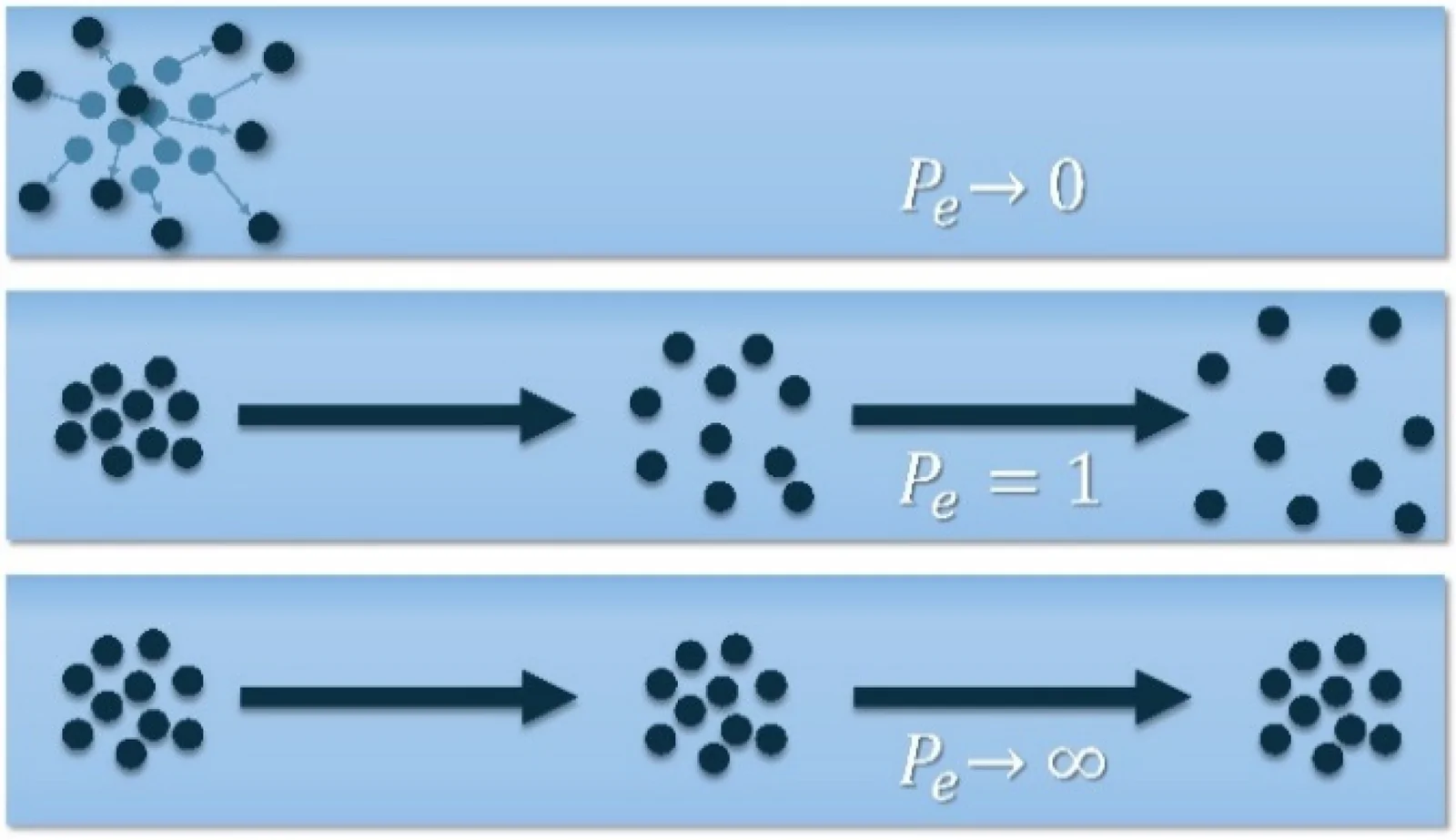
Visualisation of different mass transport regimes described by the Peclet number.

These factors allow for a comparison to be made between different reactors and reaction set-ups. The axial dispersion model was used for fitting the *E*(*θ*) based on eqn (2).^30^ Non-linear least squares analysis was performed to obtain vales for *τ*_r_ and *P*_e_. Obtaining these values has been covered in previous publications,^31–34^ in this present case we developed both Python and MATLAB scripts for this purpose, both of which can be found in ESI† in addition to exemplars of the relevant fitted data.2
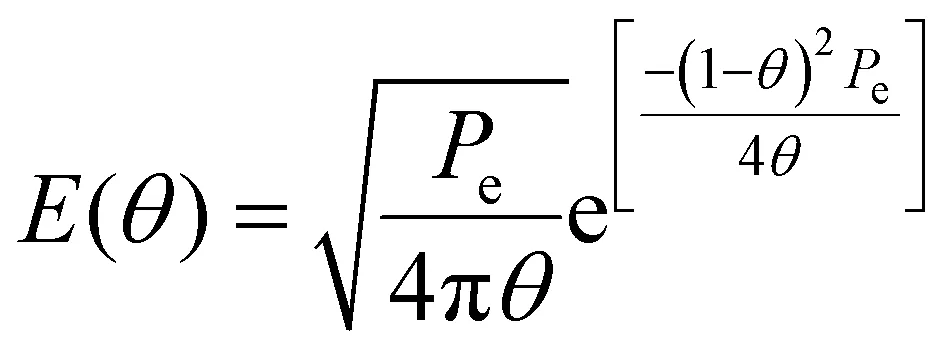
where *P*_e_ = Peclet number, 
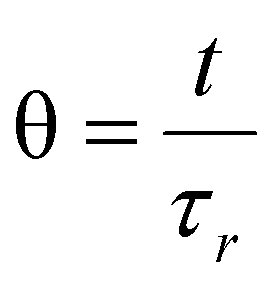
 and *τ*_r_ is the average residence time of the reactor (Table 1).

**Table 1:** *P*_e_ and *τ*_r_ of a 10 mL tubular reactor at flow rates of 0.125 to 2 mL min^−1^

Reactor volume (mL)	Flow rate (mL min^−1^)	Average residence time (mins)	Peclet number
10	2	5.27	94
10	1	10.4	129
10	0.5	20.7	137
10	0.25	41.1	194
10	0.125	81.7	370
20	2	10.4	192
20	1	21	290
20	0.5	41	306
20	0.25	83	421
20	0.125	166	821

**Fig. 8:**
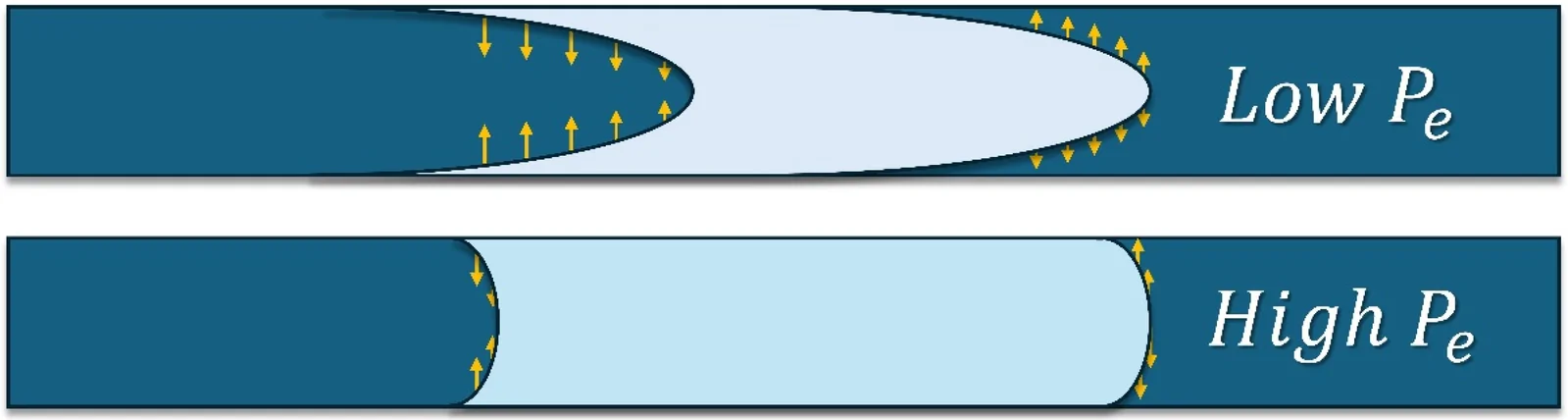
Visualization of the parabolic flow profiles of a fluid system at different Peclet values.

For this reactor a clear trend emerges with the *P*_e_ increasing as flow rate decreases, this is as expected and is caused by the decrease of the parabolic velocity of the regime as the flow rate decreases (Fig. 8). This supports the qualitative assessment from the normalized RTD traces. One key observation of this dataset is the very different mixing regimes within separate reactors when *τ*_r_ are similar. For example, when the average residence time (*τ*_r_) = 20 minutes for both the 10 mL and 20 mL reactors, we see an increase of Péclet number by a factor of 2 indicating increased mixing. This study of residence times and Péclet number can be instrumental in deciding which flow regime best suits the specific chemistry of interest when performing a chemical transformation in continuous flow.

It must be noted that these tracers typically do not perfectly reflect the polymerization regime, as a single monodisperse small molecule has been used in lieu of a polymer with a relatively broad molecular mass distribution By definition in a polymer there are often many hundreds if not thousands of individual discrete molecules as quantified by the dispersity, which also confer increases in viscosity due to increase in hydrodynamic volume. This approach does, however, give a straightforward approach to directly compare reactor and flow rate conditions, and give the chemist greater insight into the fluid dynamics within their reaction volume.

## Online and real time monitoring of batch reactions

Chemical transformations are most often carried out in batch processes in flasks or stirred tank reactors in both laboratories and on larger scale industrial processes. It is common for kinetic studies to be conducted in this way, with aliquots taken at defined intervals and interrogated offline by appropriate analysis. This approach is valuable and relatively simple to accomplish for reactions that occur in a reasonable time frame of typically up to a few hours.

**Fig. 9:**
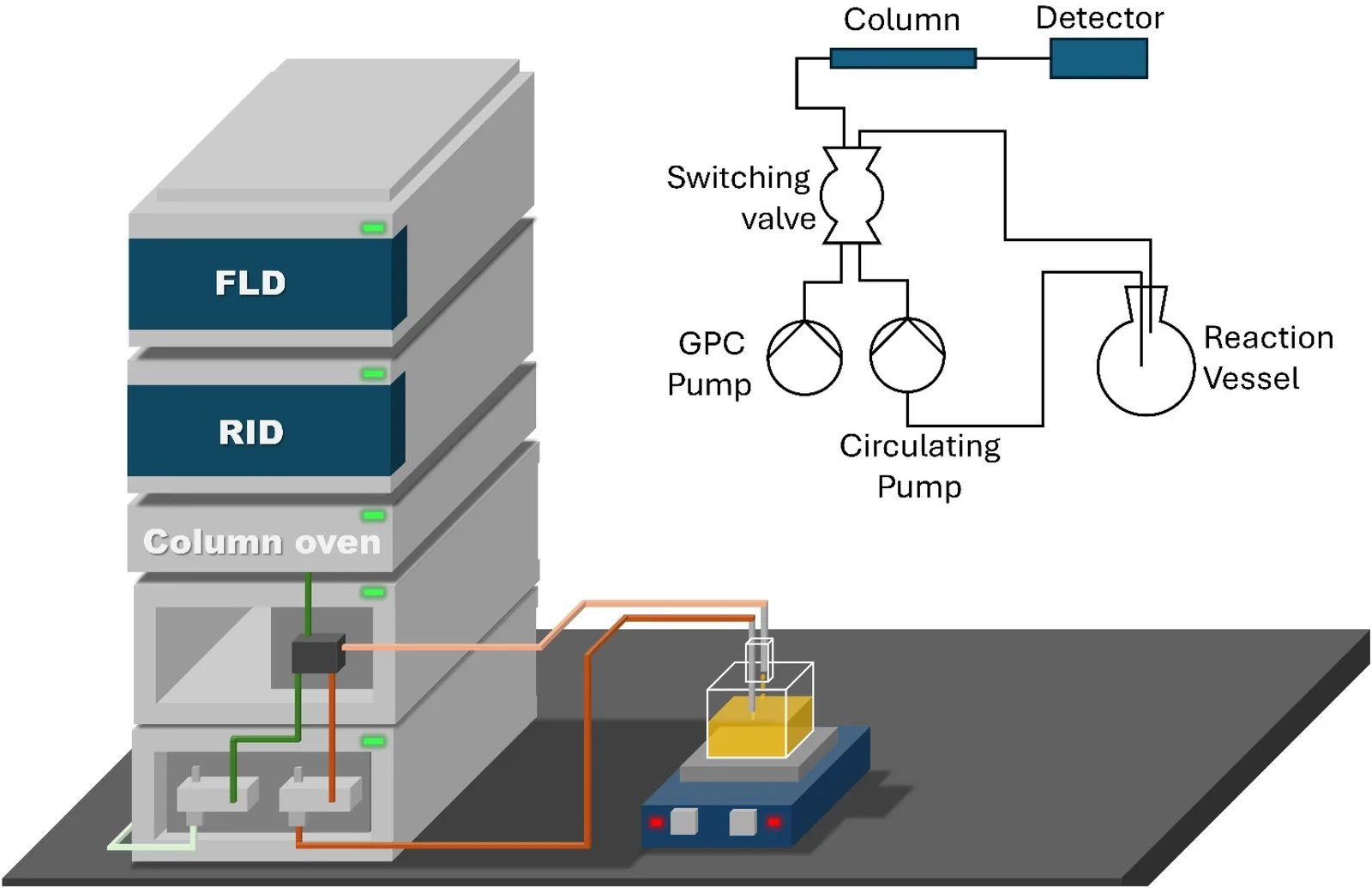
Batch polymerization monitoring of a RAFT polymerization of MA moderated with PABTC initiated with AIBN.

To accomplish batch reaction monitoring using HPLC equipment, an Agilent binary pump, autosampler, RID and FLD were employed as part of the online GPC system. A binary pump is essential as one pump is required for supplying the eluent, and a second for circulating the reaction mixture (Fig. 9). An “OpenLabs” method was written for this experimental set-up, setting each pump to 1 mL min^−1^ and collecting data from the RID for analysis.

RAFT polymerization of methyl acrylate (MA) was carried out using 2-(butylthiocarbonothioylthio)propanoic acid (PABTC) as the RAFT agent and AIBN as initiator in 1,4-dioxane as solvent, in a single necked round bottom flask. The mixture was heated to 70 °C and circulated through the switching valve by the dual-piston HPLC pump at 1 ml min^−1^. The total volume of the circulating loop including the sample loop within the valve was 0.5 mL, corresponding to a circulation time of 30 seconds. It is noted that this methodology could be employed with any traditional batch experiment at any scale.

Throughout the course of the reaction, samples for GPC were taken from the sample loop every 15 minutes for 20 hours giving a total of 80 chromatograms throughout the reaction. It is important to note with this setup that during the injection process 20 μL of eluent is added to the circulating reaction mixture. In this case the eluent was THF and the reaction solvent 1,4-dioxane and no adverse effects were noted on the product formed. However, additional care must be taken if this approach is used for techniques that require anhydrous conditions or are sensitive to minor changes in the solvent make up. The effect of dilution is minimal in this experiment, with only 1.6 mL of eluent added to the reaction over the period of 20 hours, this does not affect the overall volume of the reaction as an equal amount of solution is removed as is replaced.

**Fig. 10:**
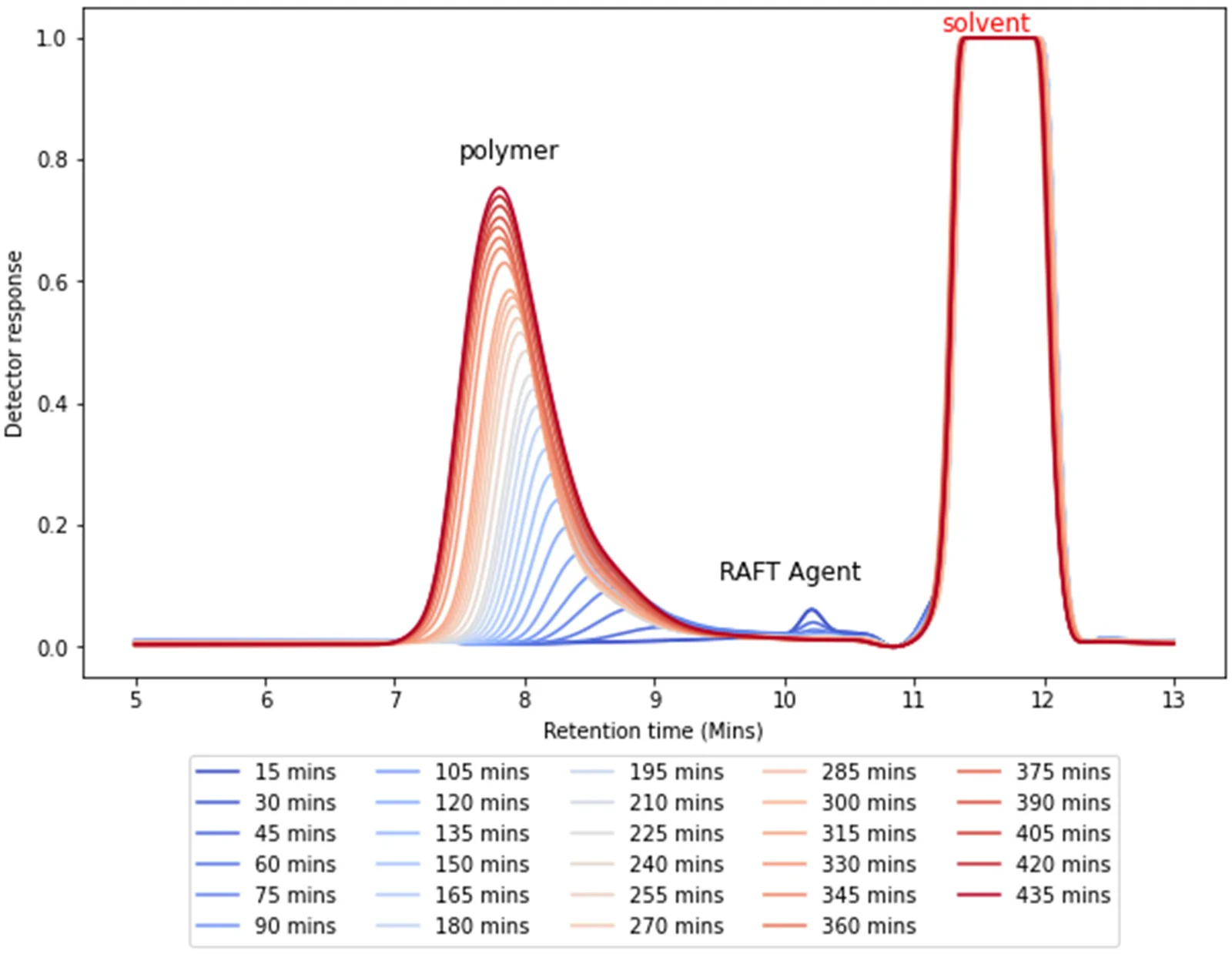
Overlayed normalized GPC traces from RAFT polymerization of MA moderated with PABTC and initiated with AIBN at 70 °C ranging from initiation to *T* = 20 hours at intervals of 15 minutes.

**Fig. 11:**
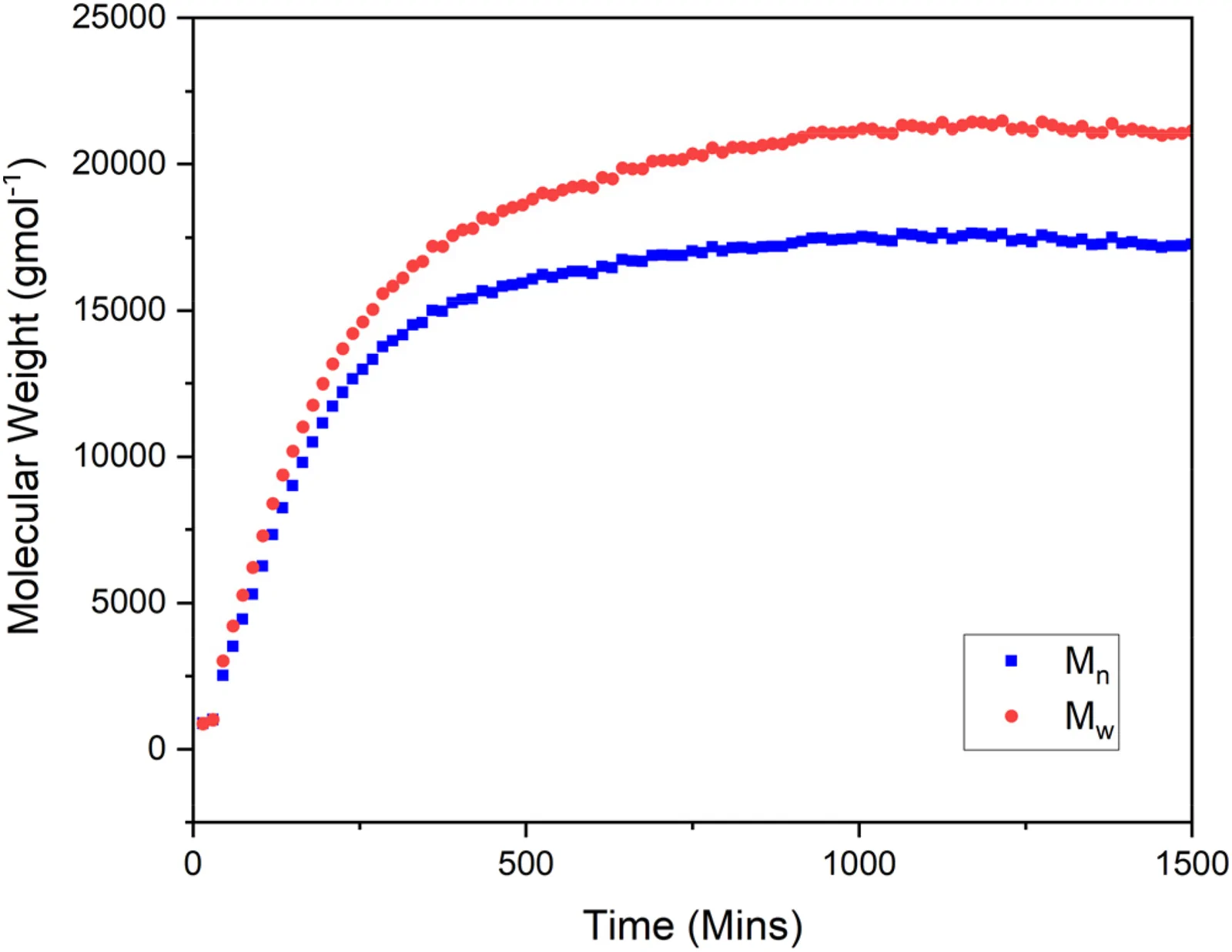
Online GPC molecular weights, *M*_n_ (red) and *M*_w_ (blue), for the batch monitoring of the RAFT polymerisation of MA moderated with PABTC and initiated with AIBN ranging from initiation to *T* = 20 hours.

The overlayed GPC chromatograms (Fig. 10) demonstrate an increasing mass over reaction time (Fig. 11) and a corresponding increase in peak area which directly correlates with monomer conversion (monomer conversion % = 100 − polymer % produced) as this is the total amount of light refracted from the polymer as the DRI is a true concentration detector of the repeat unit in the polymer. This level of temporal resolution is high for polymer reaction monitoring, even though the column with longest acquisition time of those tested were used. Additional monitoring demonstrates that this level of detail is trivial to acquire with the modified HPLC set up, and experiments can be run multiple times per day or for multiple days without issue.

A feature of note in these GPC chromatograms is the large solvent/monomer peak at between 11 and 12.2 minutes. As samples are taken directly from the reaction mixture without dilution, this large peak is inevitable but largely irrelevant due to the nature of the GPC separating the analyte by size in solution. When the reaction produces low molecular weight MMA dimers and trimers (approximately 200 and 300 g mol^−1^) produced through CCTP (Fig. 16) as discussed later, the solvent peak does not interfere with effective polymer analysis however care needs to be taken when looking at relatively low molecular weights. Another interesting feature of these traces is the small peak eluting at approximately 10.3 minutes. This signal has been attributed to the PABTC RAFT agent, a more detailed plot showing this RAFT agent decay can be found in the ESI.† The peak can be seen to decrease as the RAFT agent is consumed and is fully consumed by *T* = 45 min. From these traces, both *M*_n_ and *M*_w_ can be obtained to show the evolution of the molecular weight over time (Fig. 11). These spectra were then processed using a custom GPC-analysis Python program to obtain molecular weight distribution (dispersity) values. In this case a PMMA calibration was used, and the entire elution volumes (5–9.8 minutes) integrated. This range was chosen to exclude the possibility of misrepresenting the data if any large species had been produced. The cut-off of 9.8 minutes was chosen to minimize the overlap with the peak caused by the RAFT agent. For the 1^st^ and 2^nd^ data point only very low molecular weight species had been produced with significant overlap form the RAFT agent, therefore the calculations were manually conducted with a forced integration region of 9.0 to 9.8 min.

**Fig. 12:**
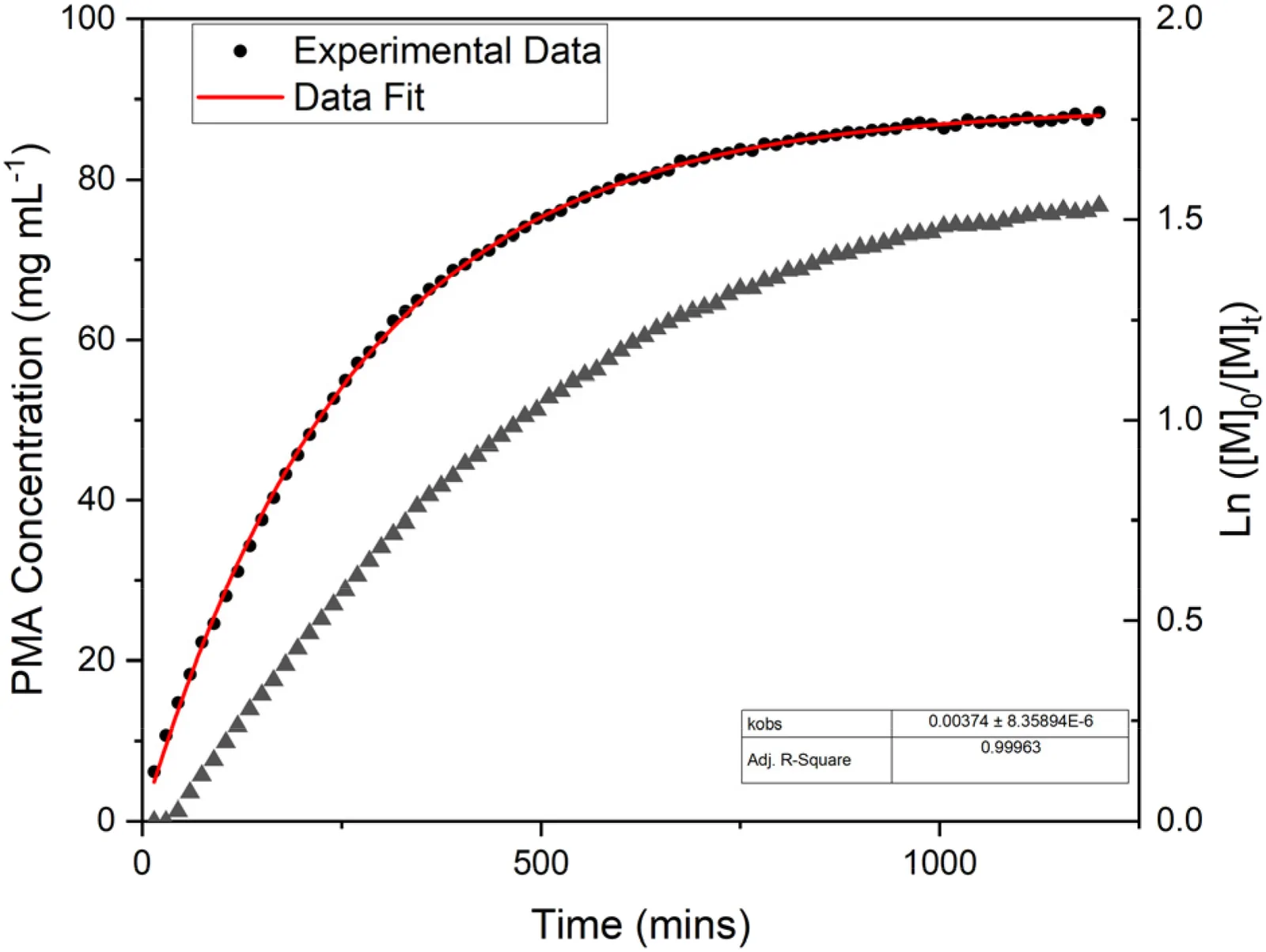
Concentration of PMA in solution for the batch monitoring of the RAFT polymerisation of MA moderated with PABTC initiated with AIBN from initiation to *T* = 20 hours.

The absolute concentration of the polymer is also obtained directly from the area of the polymer peak from the DRI detector (Fig. 12). This is set by using the detector constant of the RI and obtained using eqn (3).3
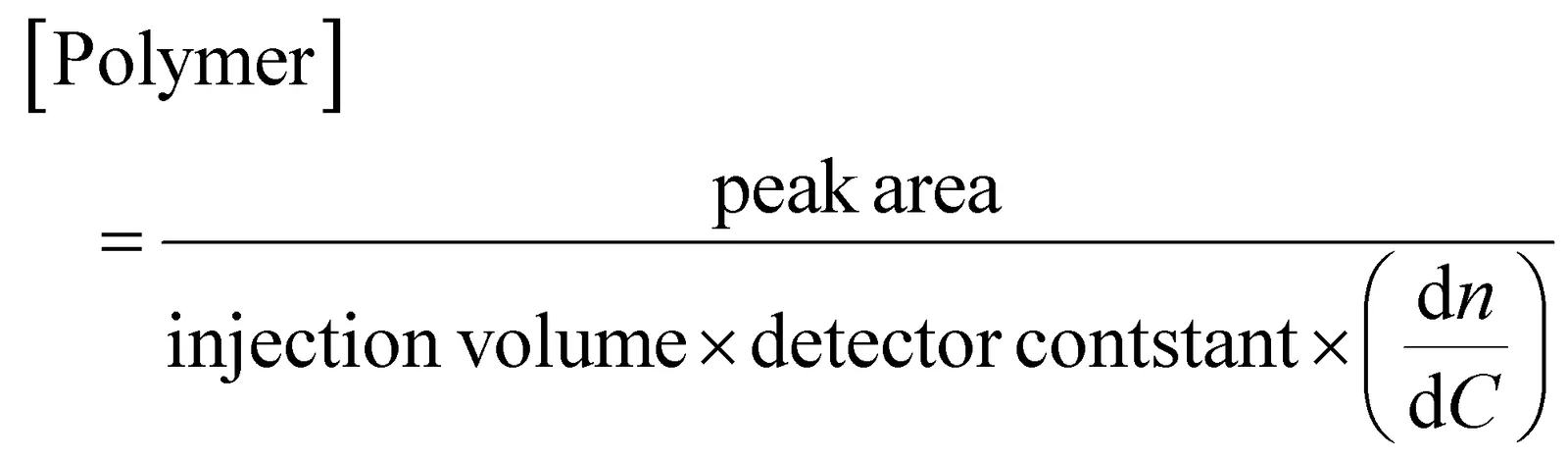


**Fig. 13:**
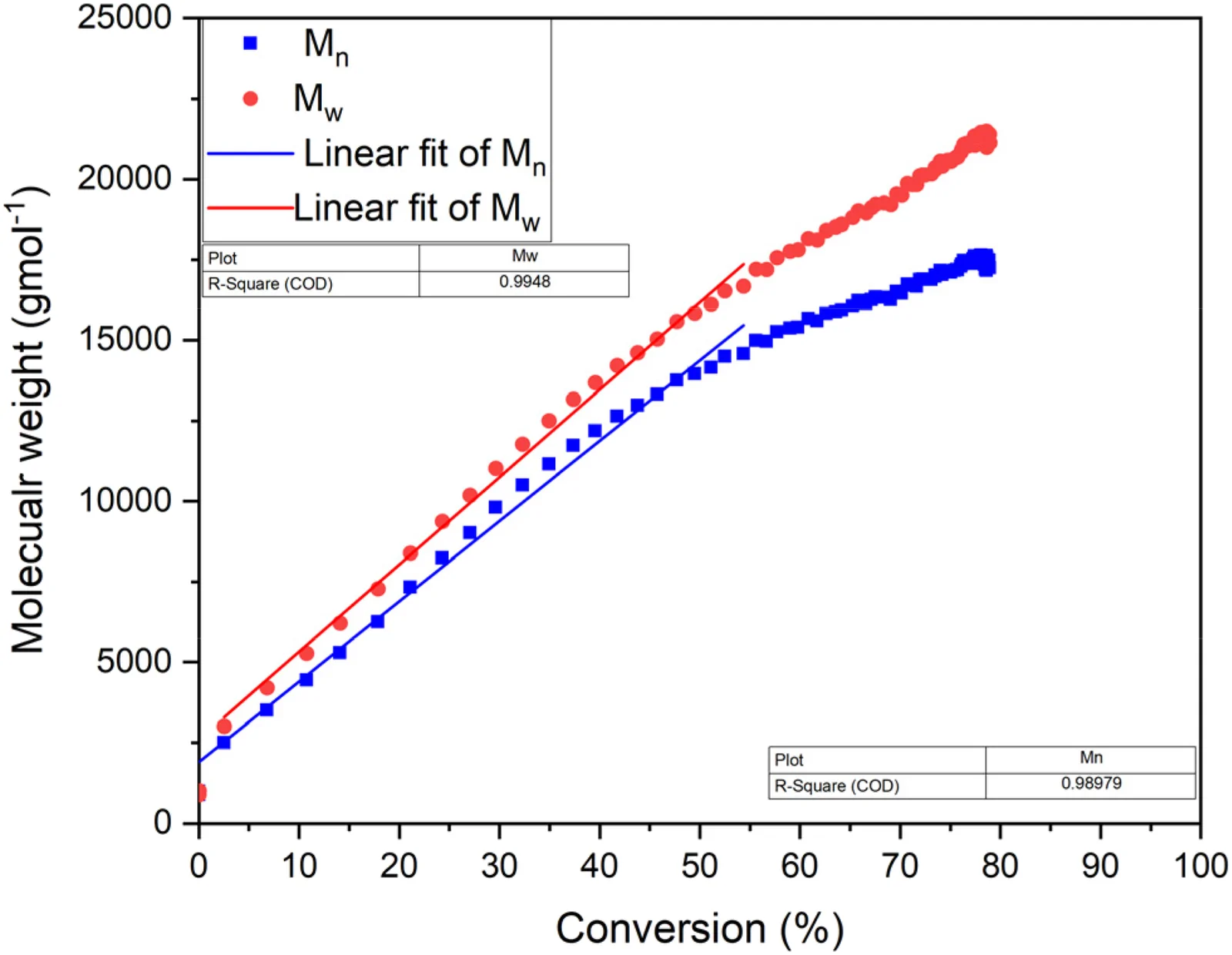
Molecular weights (*M*_n_ – red and *M*_w_ – blue) for the batch monitoring of the RAFT polymerisation of MA moderated with PABTC and initiated with AIBN.

This also allows for percentage monomer conversion calculations by comparison to the theoretical maximum concentration of each reaction. From experimentally determined concentration the rate of reaction can be determined by fitting appropriate models to these data sets. A 1^st^ order fit of the conversion data (Fig. 12) gives a rate constant of *k*_obs_ = 0.0037 s^−1^ with an error of 8.7 × 10^−6^ s^−1^, *R*^2^ = 0.9998 in this case, over the first 60% monomer conversion.^35^ The first order rate plot (Fig. 12) shows significant termination at just over 500 minutes (conversion is approximately 70% at this point) which is consistent with the decrease in the rate of growth of the molecular weight (Fig. 13).

**Fig. 14:**
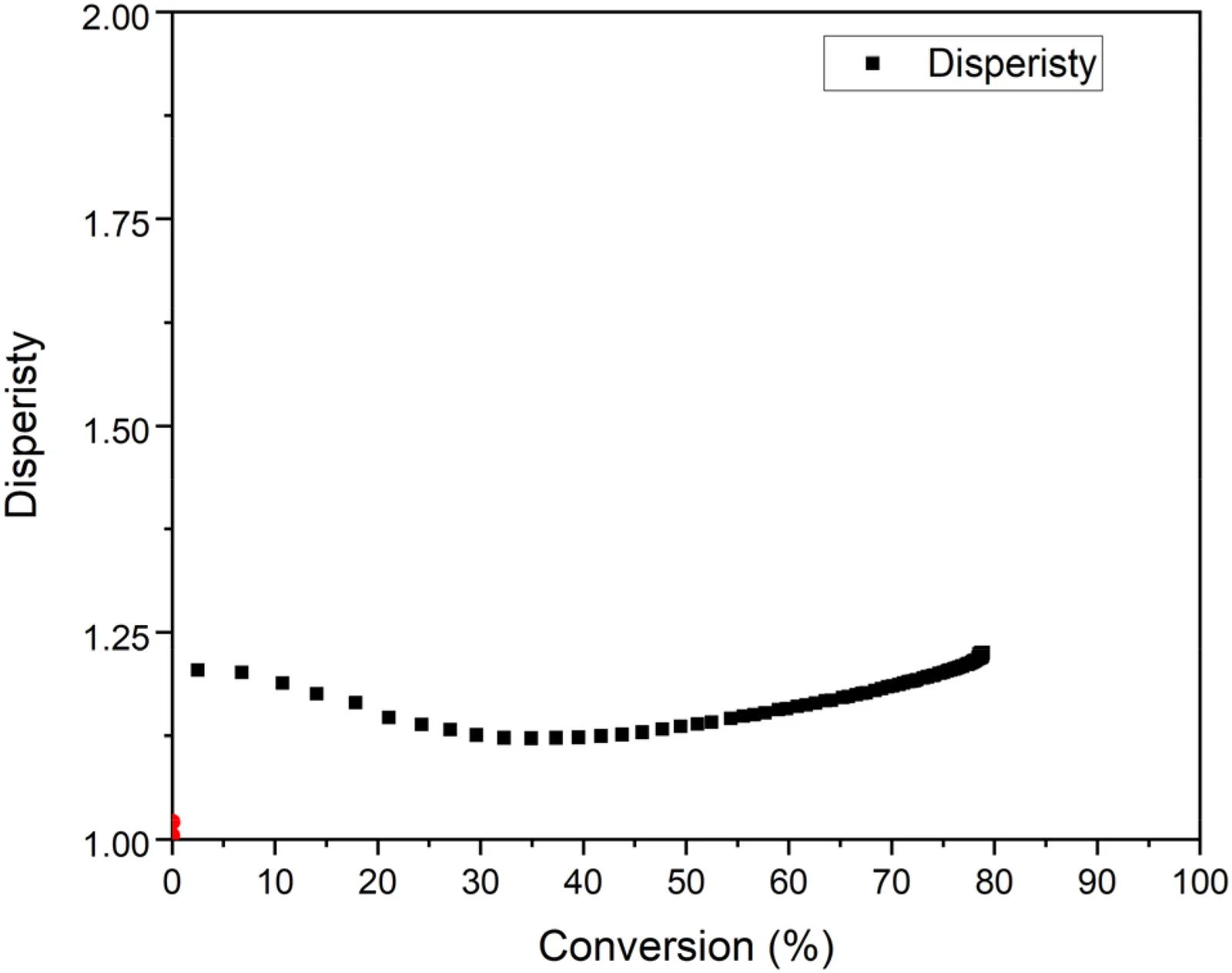
Dispersity as a function of conversion for the RAFT polymerisation of MA moderated with PABTC initiated with AIBN.

The fit for this plot is good up to the point where radical–radical termination reactions would be expected in RAFT of acrylates under these conditions, highlighting the power of the online GPC approach when applied to monitoring batch reactions. It is also straightforward to monitor both the increase in average mass and dispersity as the reaction progress (Fig. 13 and 14). This data shows a relatively good linear increase in the *M*_n_, as would be expected for a living polymerisation with a final *R*^2^ values = 0.99 for both *M*_w_ and *M*_n_. There is some deviation from the linear fit for both *M*_n_ and *M*_w_, this is due to the nature of the radical process being performed.

Interestingly there is some variance in the dispersity as the polymerisation progresses until it reaches its final conversion of 80% and final dispersity = 1.2. It is important to note that in this case the first two data points overlapped with the peak from the RAFT agent and had very low conversion, <0.1%, therefore the fits shown in Fig. 12 and 13 have had these points excluded.

## Determination of chain transfer constants from CCTP in continuous flow

**Fig. 15:**
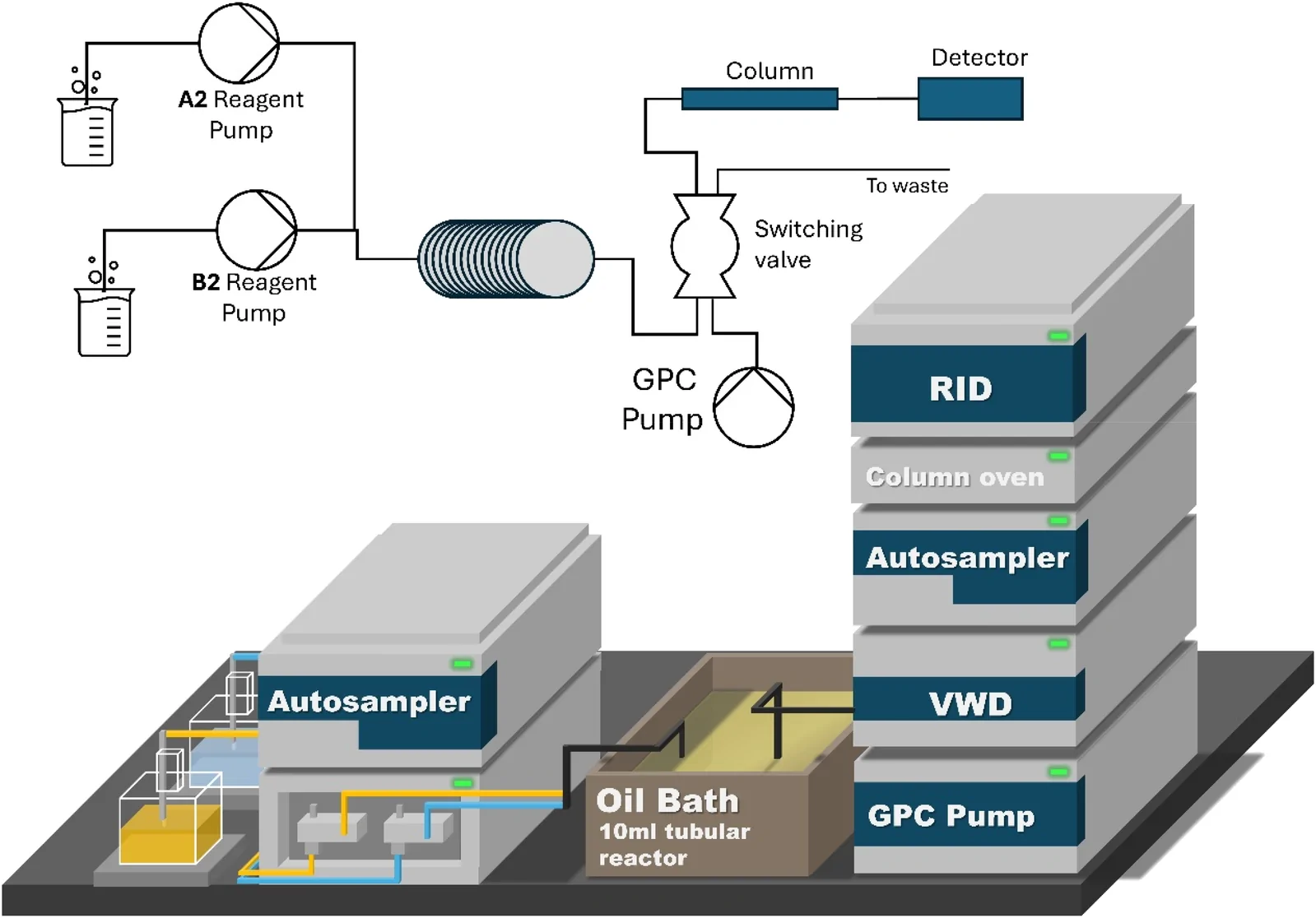
Continuous flow polymerisation monitoring of the CCTP polymerization of MMA using CoBF as catalytic chain transfer agent (CCTA) with AIBN as the initiator.

The ability to obtain chain transfer constants is desirable when studying and using free radical polymerization. To accomplish this, a binary HPLC pump was employed to deliver two separate streams of reagents into the flowline (Fig. 15). One stream contained monomer, solvent, initiator and CoBF catalyst (A2), the second only monomer, initiator and solvent (B2). The two solutions were then pumped and mixed at a constant flow rate over a period of 10 hours through a 10 mL tubular reactor held at 70 °C with a constant overall flow rate of 0.5 mL min^−1^, the RTD of this fluid regime has been discussed previously. The contributions of each reagent stream were altered to produce a series of mixtures with different [MMA]/[CoBF] ratios. This was accomplished by altering the percentage contribution of each pump using *OpenLabs* HPLC control software. The sequence started using a 100% contribution from A2 holding a steady state for 60 minutes, then decreasing this contribution to 90% and increasing the monomer/solvent line B2 to 10%, thus increasing the [MMA]/[CoBF]. This continued in 10% increments until a continuous stream of B2 was used. This produced a set of steady-state reaction conditions with [CoBF] content varying from 10 ppm to 1 ppm (Table 2).

**Table 2:** Reaction conditions for the continuous flow polymerisation monitoring of the CCTP polymerization of MMA using CoBF as catalytic chain transfer agent (CCTA) with AIBN as the initiator

	AIBN (mg)	Flow rate (ml min^−1^)/residence time (min)	Temp. (°C)
Group A	250	0.5/20	70
Group B	250	0.25/40	70
Group C	250	0.5/20	80
Group D	125	0.5/20	70

**Fig. 16:**
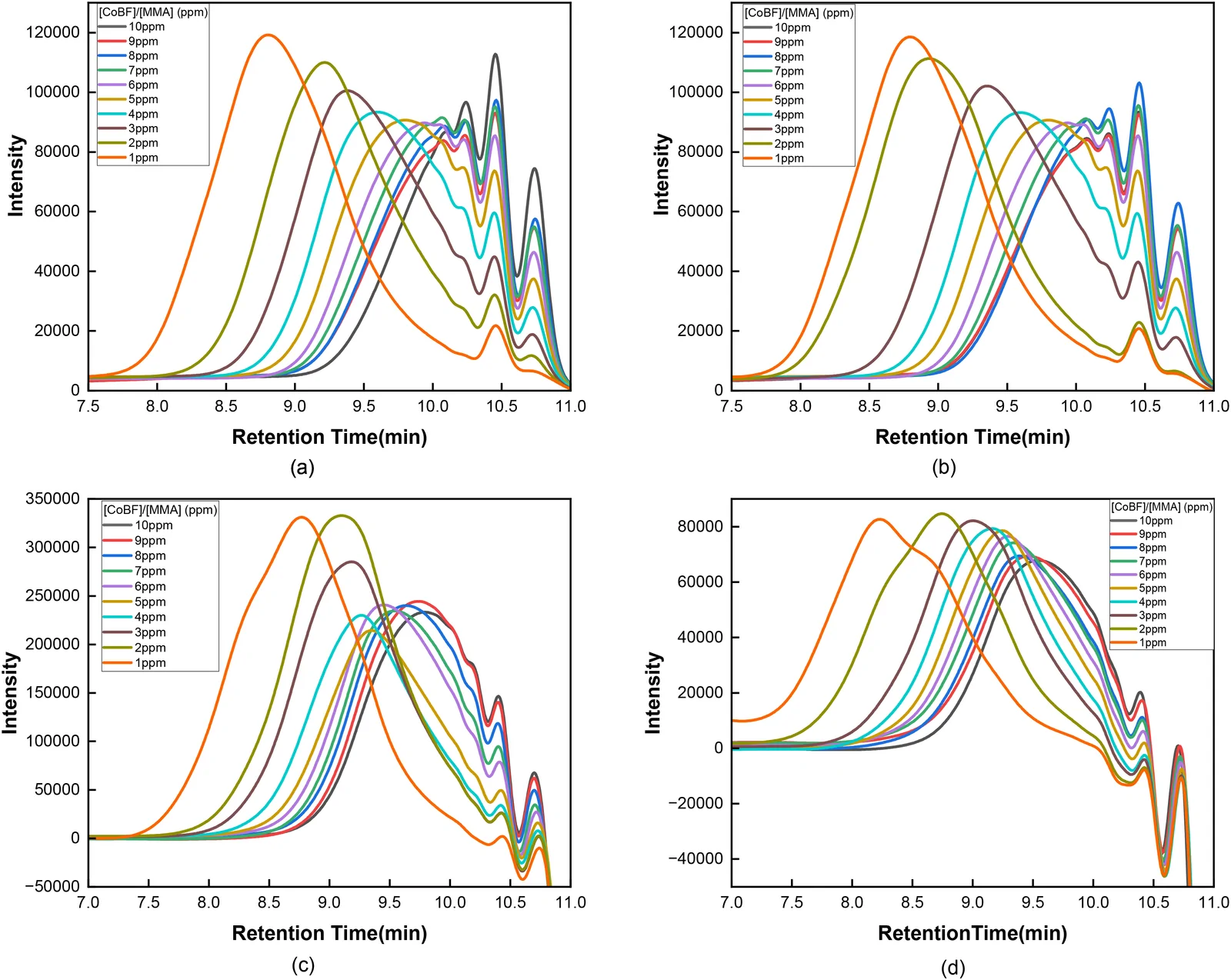
GPC traces of four experiments undertaken to determine the chain transfer constant (*C*_s_) of MMA in toluene by CCTP using AIBN as initiator and CoBF as catalyst. Group A (a) – 250 mg AIBN, *τ*_r_ = 20 min 70 °C, Group B (b) – 250 mg AIBN, *τ*_r_ = 40 min 70 °C, Group C (c) – 250 mg AIBN, *τ*_r_ = 20 min 80 °C, Group D (d) – 125 mg AIBN, *τ*_r_ = 20 min 70 °C.

Each of the ten steady-state conditions were sampled using online GPC analysis. Sampling was accomplished using a continuous sequence of 40 identical GPC methods. This provided four independent GPC traces for each set of reaction conditions, Fig. 16(a). We observed an increase in *M*_n_ and *M*_w_ as the [CoBF] decreases with *Đ* increasing from 1.7 to 2.7 as [CoBF] reaches zero as would be expected. This is due to at lower molecular weights the distribution is narrowed at low molecular weight as the low limit is monomer and thus the distribution has *M*_n_ artificially high.^36,37^

Following from these initial results, further experiments were conducted varying (a) reaction time (Fig. 16b), (b) temperature (Fig. 16c), and (c) initiator concentration (Fig. 16d). Decreasing the combined flow rate to 0.25 ml min^−1^ (residence time = 41 min, *P*_e_ = 194.46) had the effect of increasing the *M*_n_ and *M*_w_ of the polymers produced and increasing the overall conversion of the reaction, Fig. 16(b). This is consistent with expected results from increased reaction time. In decreasing the flow rate, effective mixing was also reduced, further detailed investigations into the effect of mixing on CCTP products in flow have been the focus of previous work.^28^

**Fig. 17:**
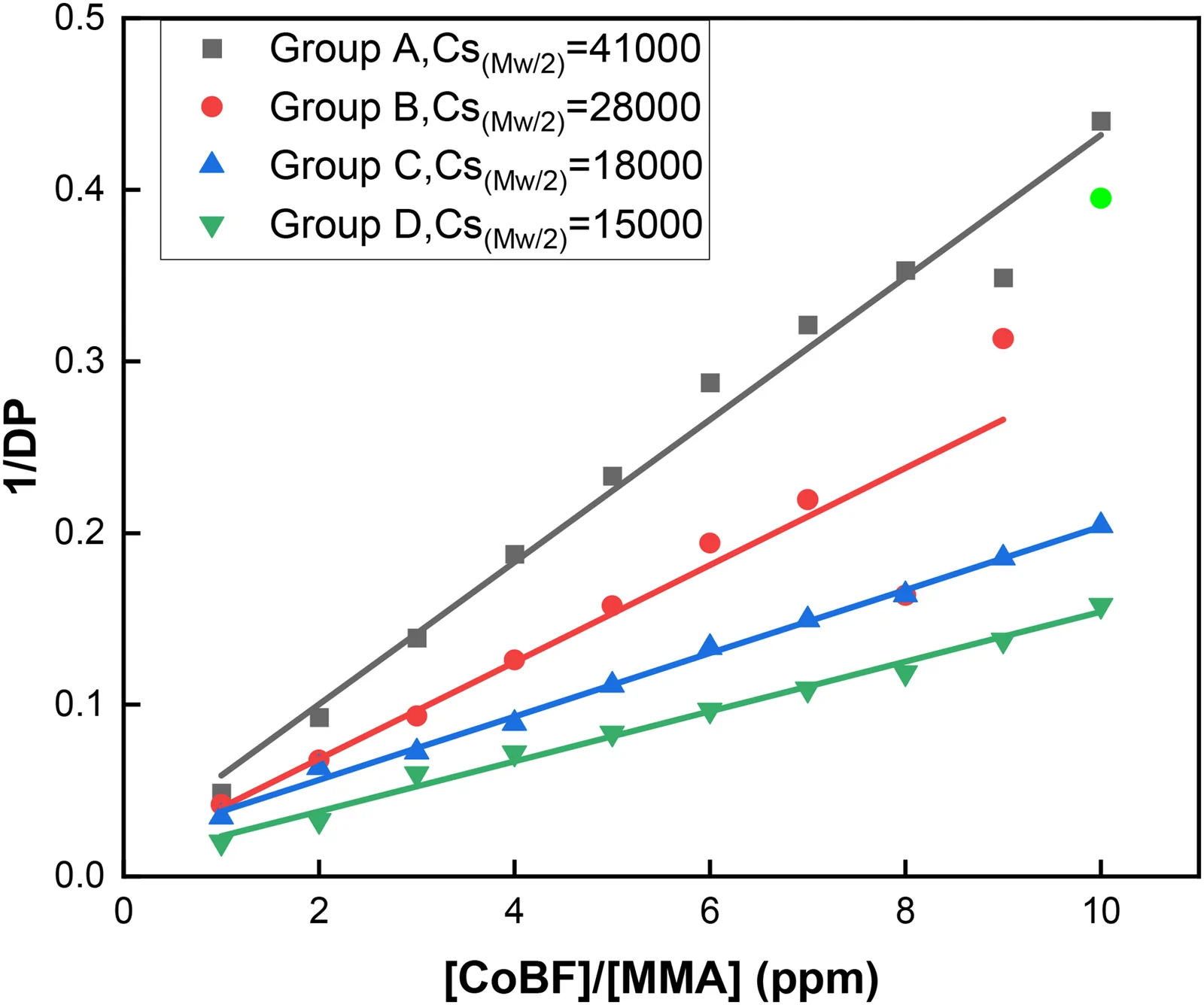
Mayo plots for the in-flow determination of *C*_s_ of four experiments with DP calculated from *M*_w_/2. Group A (Black) – 250 mg AIBN, *τ*_r_ = 20 min 70 °C, Group B (Red) – 250 mg AIBN, *τ*_r_ = 40 min 70 °C, Group C (Blue) – 250 mg AIBN, *τ*_r_ = 20 min 80 °C, Group D (Green) – 250 mg AIBN, *τ*_r_ = 20 min 70 °C.

Increasing the temperature from 70 °C to 80 °C, Fig. 16(c) gave an increase in conversion and produced a both higher *M*_n_ and *M*_w_. The effect of a lower concentration of AIBN Fig. 16(d) was also investigated. The masses of the produced polymers are higher than that of set (a) with *Đ* between 1.3 and 1.6. As *M*_n_ can be very sensitive to baseline and dispersity fluctuations, and *M*_w_ is more sensitive to high molecular weight fractions and less affected by baseline fluctuations, we used *M*_w_/2 to calculate chain transfer constant (*C*_s_) *via* the construction of Mayo plots (Fig. 17) giving values for *C*_s_.^38^ In order to be able to compare chain transfer constants in CCTP, or other types of chain transfer, it is noted that it is assumed the [Monomer]/[CTA] remains constant and as reagents are consumed in the reaction accurate chain transfer constants need to be measured at very low conversions usually <5%. In CCTP monomer is consumed but the chain transfer agent is regenerated as a catalyst. However, the CCTA is, in reality, destroyed during the reaction, by peroxide radicals for example. Thus, Group B is measured at higher conversion than Group A as the reaction is carried out for longer and there is a significantly higher concentration of radicals, and hence rate in Group C, due to the higher temperature. It is more difficult to explain the lower *C*_s_ value observed for Group D and is possibly due to a higher ingress of air/oxygen in this particular experiment and shows the importance of the integrity of the many tubing joints and different lengths of tubing in the equipment.

The ability to obtain chain transfer constants from the Mayo equation with only one reaction demonstrates the utility of the modified HPLC system using simple HPLC pump control software available for any binary HPLC pump system to construct complex experimental methods without needing to code.

## RAFT polymerisation in continuous flow

**Fig. 18:**
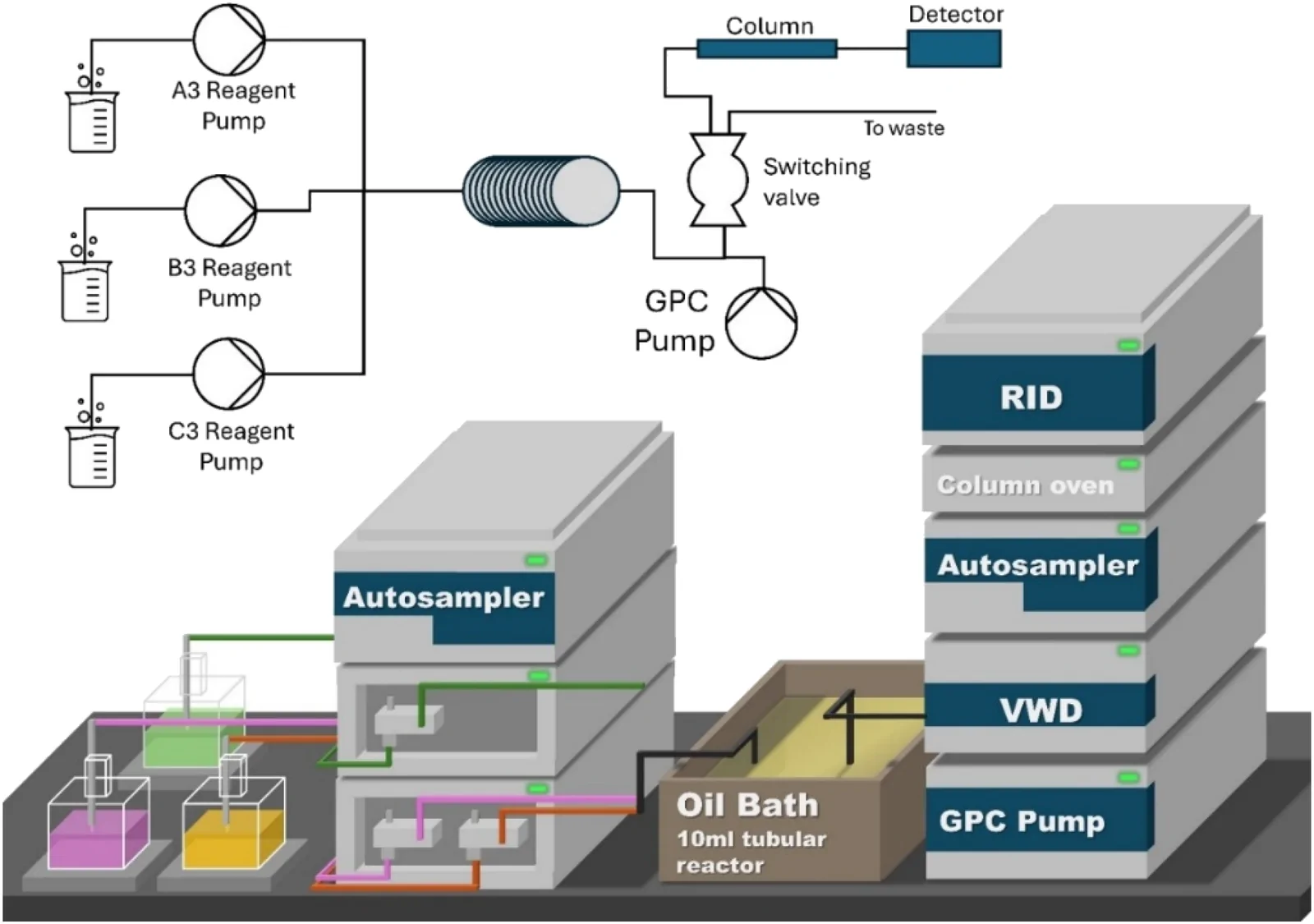
Continuous flow polymerisation monitoring of the RAFT polymerization of DMA using PABTC RAFT agent and AIBN as the initiator.

To show the flexibility of modified HPLC systems, an alternative configuration was used. Two Binary Pump HPLC modules were employed to deliver 3 *different* reagent streams, Fig. 18. The first (a) consisted of only monomer and solvent (**A3**), the second (b) monomer, solvent and RAFT agent (**B3**) and the final stream (c) contained initiator dissolved in solvent (**C3**).

**Fig. 19:**
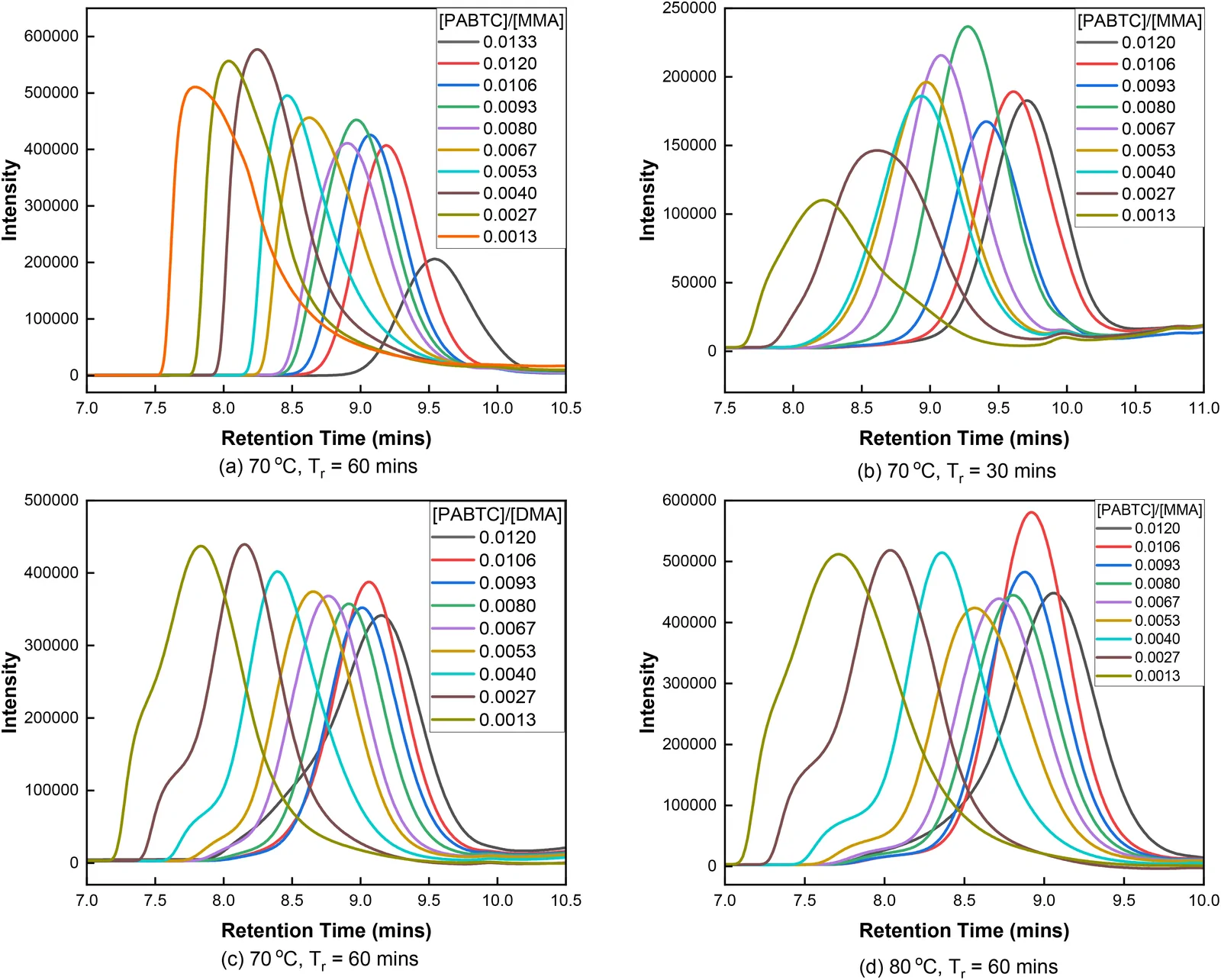
GPC traces of four experiments undertaken of DMA in 1,4-doxane by RAFT using ACVA as initiator and PABTC as catalyst. (a) 70 °C, *T*_r_ = 60 min; (b) 70 °C, residence time 30 minutes; (c) 70 °C, residence time 60 minutes; (d) 80 °C, residence time 60 minutes.

The three reagent streams were mixed and combined and flowed together through a 10 ml tubular reactor held at 70 °C at a combined rate of 0.167 mL min^−1^ with a residence time = 60 minutes. The reagent stream containing the initiator, ACVA, was pumped at a steady rate = 0.056 mL min^−1^. This flow line was joined with a combination of the other two reagent streams. As before these streams were modified in increment in order to change the [RAFT agent] this was accomplished in an identical manner to the investigations of CCTP, in initially by using 90% contribution from the flow line containing monomer, solvent, and RAFT agent and decreasing by increments of 10% every 60 minutes while increasing the contribution of the flow line containing only monomer and solvent by an equal amount. The flow line was sampled every 15 minutes by the online GPC, Fig. 19.

The GPC results show a very good correlation between the experimental results and predicted values, assuming a living polymerisation (Fig. 19 and Table 3). The initial set (a) shows the effect of changing the [RAFT agent] with the higher concentration producing well defined lower molecular weight polymers, and with decreasing concentration, an increase in the molecular weight and a general broadening of dispersity. Doubling the combined flow rate to 0.334 mL min^−1^ and thus halving the residence time to 30 minutes had the predictable effect of decreasing the *M*_n_ and *M*_w_ of the polymers produced and decreasing the overall conversion of the reaction, set (b).

**Table 3:** Reaction conditions for four groups of RAFT reactions

	ACVA content (mg)	Flow rate (mL min^−1^)/residence time (min)	Temperature (°C)
A	47	0.167/60	70
B	47	0.334/30	70
C	30	0.167/30	70
D	30	0.167/30	80

Set (c), Fig. 19, shows the effect of reducing the [ACVA] present in the reaction mixture with observed masses of the produced polymers being higher than that of set (a). Compared to set (a), decreasing the initiator leads to an increase in both *M*_n_ and *M*_w_. Compared to set (c), increasing the temperature results in increased *M*_n_, *M*_w_, and conversion. Increasing the temperature accelerates the decomposition rate of ACVA which increases the polymerization rate and thus accelerates monomer conversion to polymer. The emphasis of this work is not specifically the identity of the polymers produced, or the chemistry employed, but the experimental procedure to rapidly synthesize and characterise a range of materials with little to no human involvement. In the two cases shown (CCTP and RAFT) model reactions have been used to highlight how rapidly material discovery can be conducted without the need to develop hardware and software from scratch.

## Conclusions

We demonstrate that a straightforward modification to HPLC hardware can enable online GPC monitoring of polymerisations. We report on the separatory power of a range of GPC columns, and compared their use for online monitoring, balancing temporal and mass resolution. This has then been applied to demonstrate how this can be used effectively to monitor reactions conducted in batch. Additionally, we have proceeded to show how the same hardware can be used for complex reaction screening and determine fluid characteristics of reactions conducted in flow. While there is significant literature on this topic, the work reported in this article is an easy to access approach for chemists looking to gain access to powerful flow techniques, without the significant upfront cost of new hardware, or the time cost of developing methodology from scratch. As such we hope this approach will enable the wider polymer research community to access both online monitoring techniques and in-flow techniques more easily.

While we have focused solely on polymerisation reactions in this work, there is no intrinsic reason why this approach is not additionally applicable to small molecule synthesis and the use of normal and reverse phase HPLC columns for example. There is a growing interest in self-optimisation, machine learning and A.I. integration capabilities, however, unfortunately this is not currently compatible with the techniques described above, due to instrumentation lock outs and lack of knowledge common in scientific equipment, often only allowing control of HPLC and other hardware devices from proprietary software for specific manufacturers. These lock outs are indeed frustrating and limit advances. It is our hope that the approach and methods outlined in this communication will encourage and facilitate wider participation in the growing field of reaction monitoring and highlight to instrument manufacturers the importance of working with the scientific community to develop both their hardware and software products giving open source and open access.

## Author contributions

William Pointer: conceptualization, data curation, formal analysis, investigation, methodology, project administration, resources, software, writing original draft; Rowan Radmall: writing review and editing, methodology; Owen Tooley: writing review and editing, methodology; James Town: conceptualization, resources; Dennis C. J. Haggart: data curation; Zhichun Zhai: data curation; Xiaofan Yang: data curation; Daniel W. Lester: supervision, conceptualization, resources, funding acquisition; Paul Wilson: supervision, funding acquisition; David M. Haddleton: supervision, funding acquisition, writing review and editing.

## Conflicts of interest

There are no conflicts to declare.

## Supplementary Material

PY-016-D5PY00554J-s001

## Data Availability

The datasets generated during and/or analysed during the current study are available from the authors on reasonable request.
