# Supplementary material for: Online GPC monitoring for batch and flow polymerisation reactions
Source: Polym Chem. 2025 Jun 30;16(29):3329–43. doi: 10.1039/d5py00554j (PMC13580202; doi:10.1039/d5py00554j)
Supplement: Supplementary file 1 [file PY-016-D5PY00554J-s001.pdf]

This journal is © The Royal Society of Chemistry 2025

Online GPC monitoring for batch and flow polymerisation reactions

William Pointer[a] , Rowan Radmall[a], Owen Tooley[a], James Town[b], Dennis Haggart[a], Zhichun Zhai,[a] Xiaofan Yang [a], Daniel W Lester [b], Paul Wilson [a] and David M Haddleton* [a]

[a] *Department of Chemistry, University of Warwick, Coventry CV4 7AL, United Kingdom;*

[b] *Polymer Characterization RTP, University of Warwick, Coventry CV47AL, United Kingdom*

Supporting Information

Exemplar graphs of fitted axial dispersion model to RTDs of tubular reactors.

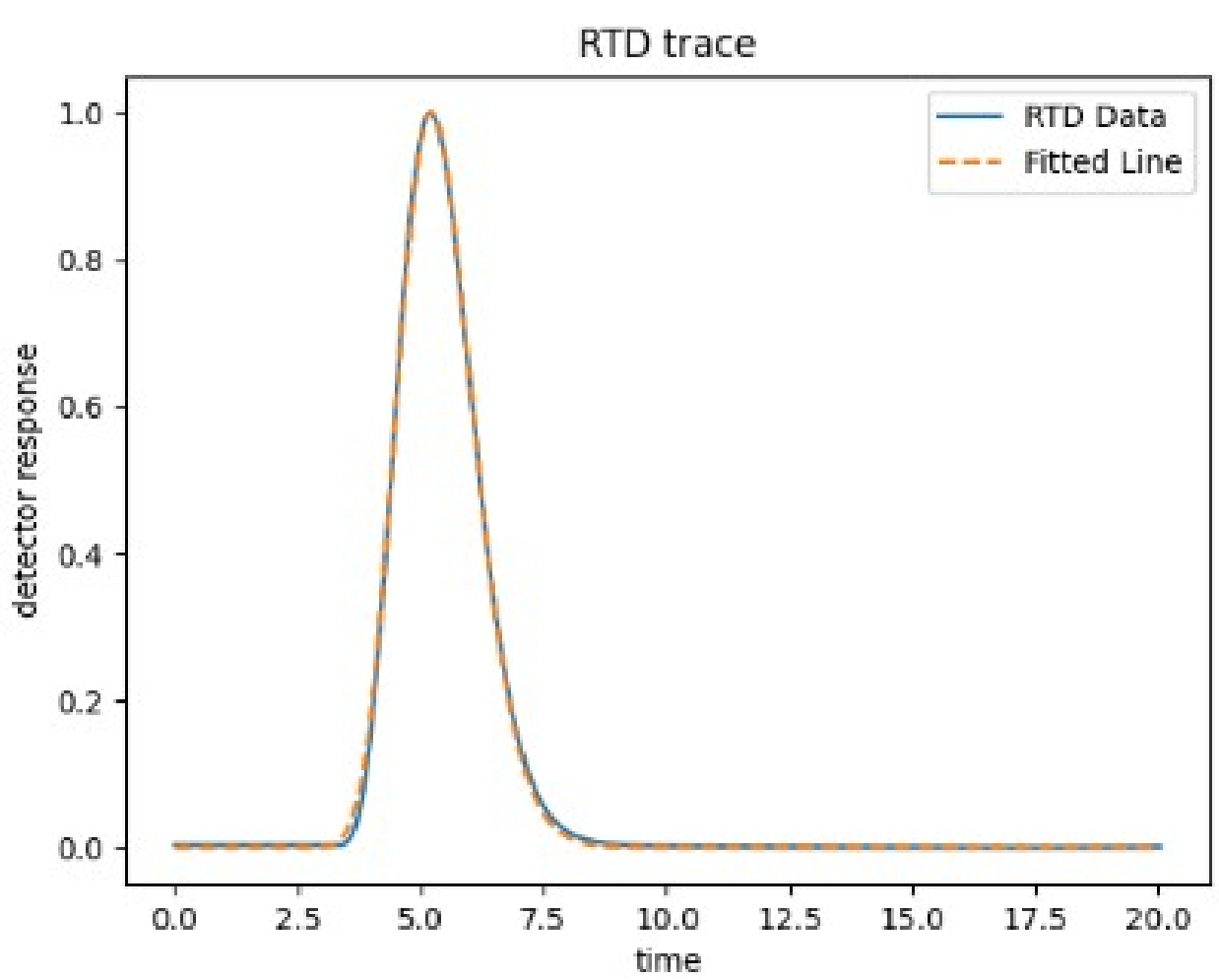

ESI-Figure 1: RTD Trace of a 10 mL 1.5 mm ID reactor tube, of THF flow rate 2 mL min^{-1} with a 100 µL toluene tracer.

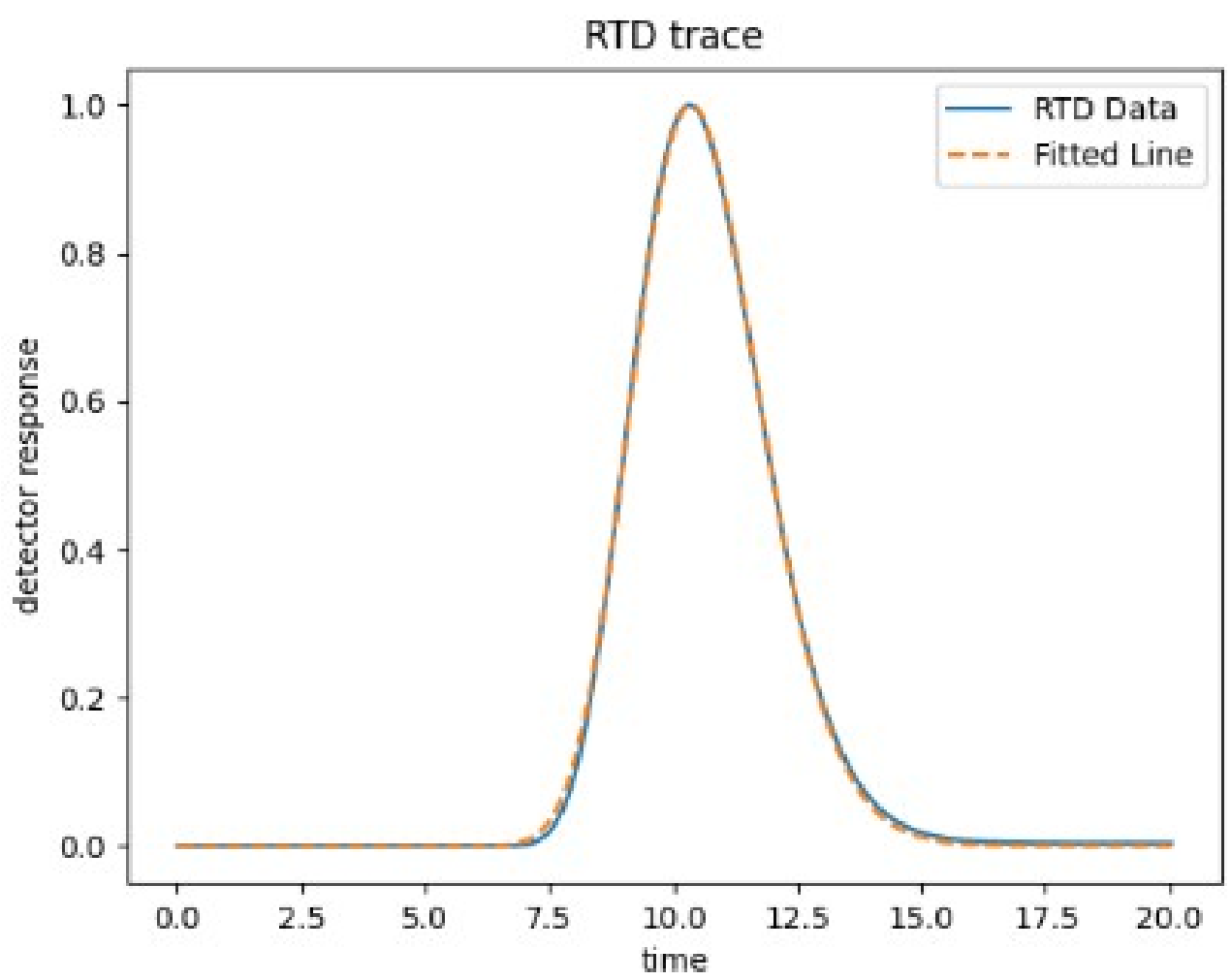

ESI-Figure 2: RTD Trace of a 10 mL 1.5 mm ID reactor tube, of THF Flow rate 1 mL min^{-1} with a 100 µL toluene tracer.

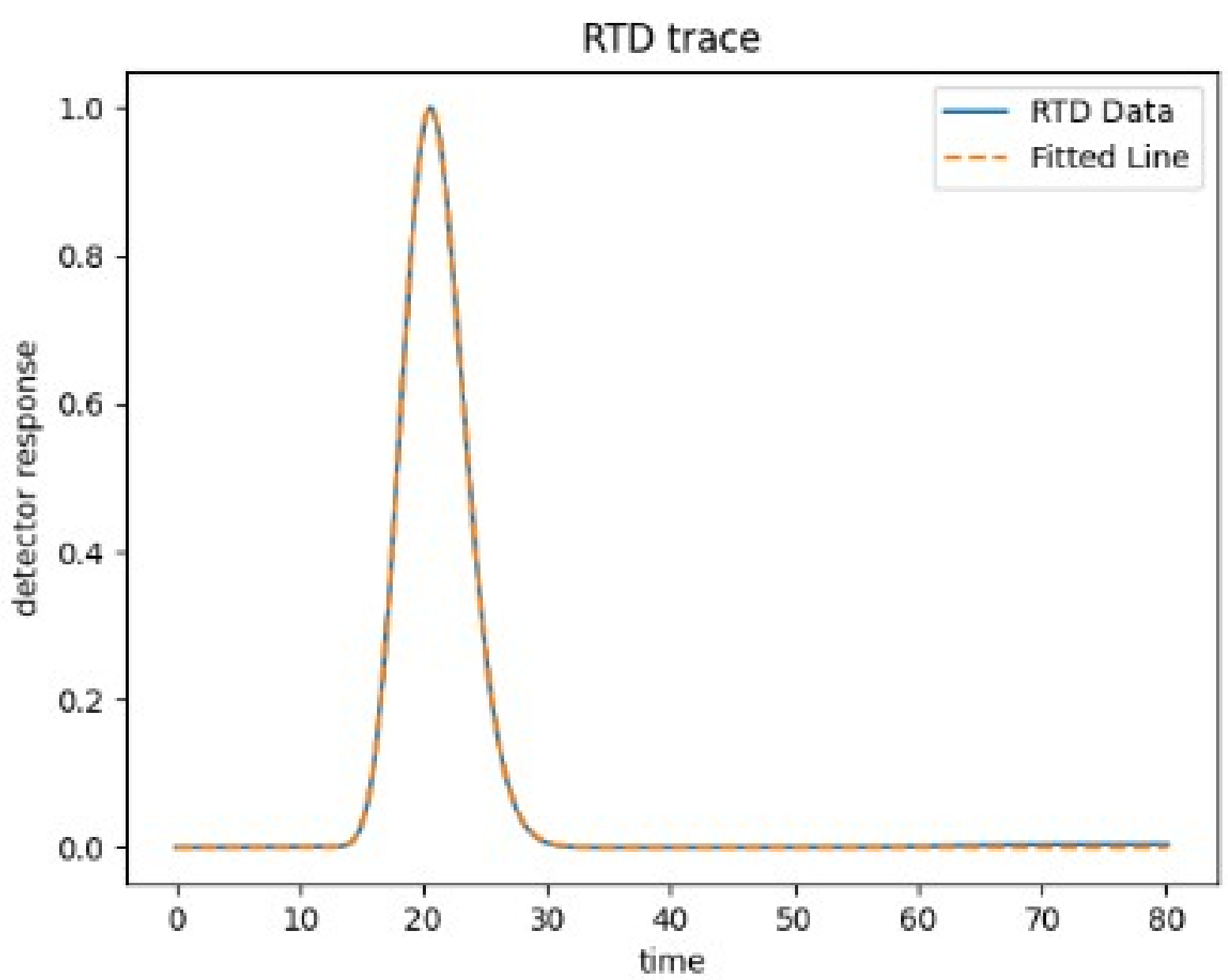

ESI-Figure 3: RTD Trace of a 10 mL 1.5mm ID reactor tube, of THF flow rate 0.5 mL min^{-1} with a 100 µL toluene tracer.

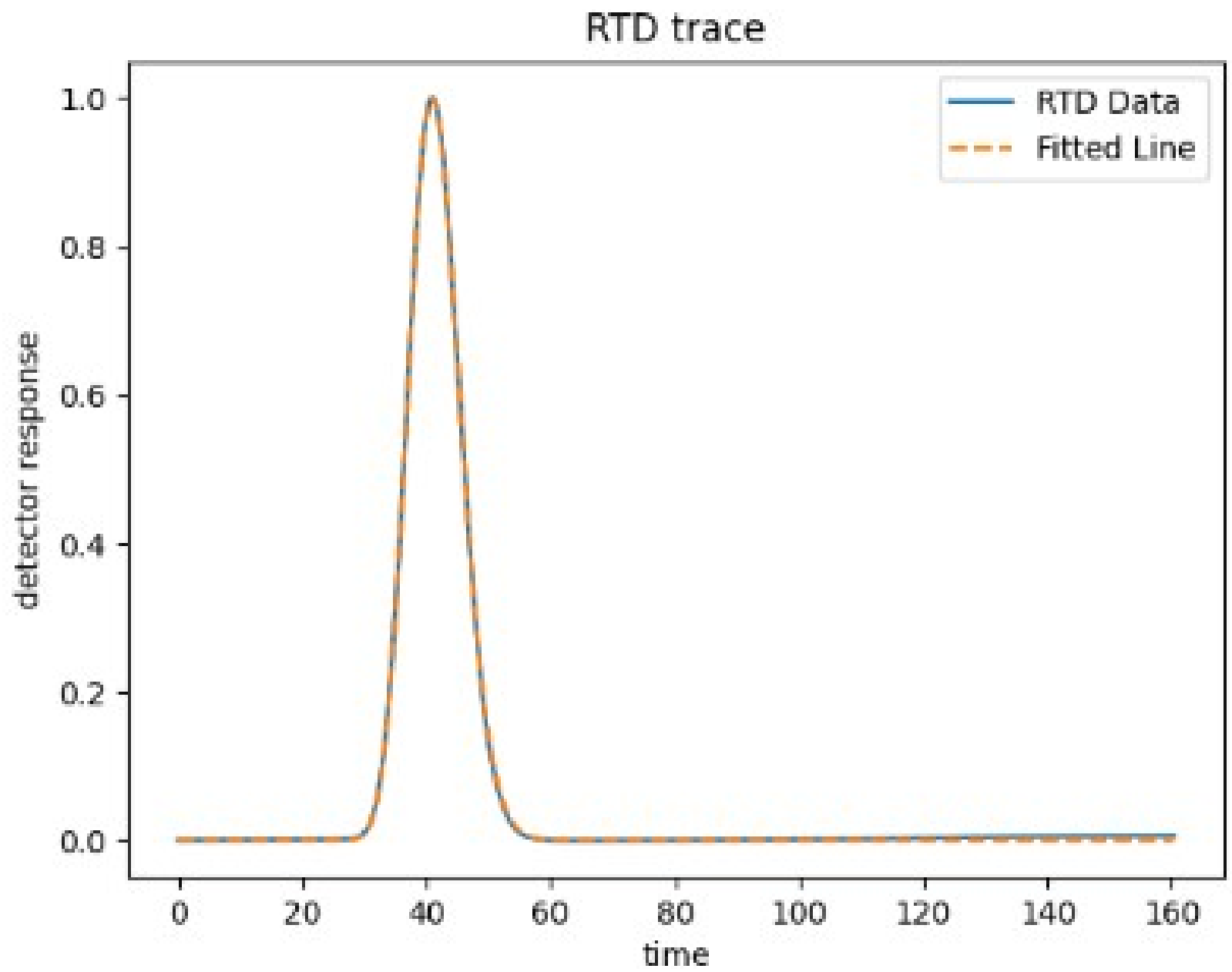

ESI-Figure 4: RTD Trace of a 10 mL 1 .5mm ID reactor tube, of THF flow rate 0.25 mL min^{-1} with a 100 µL toluene tracer.

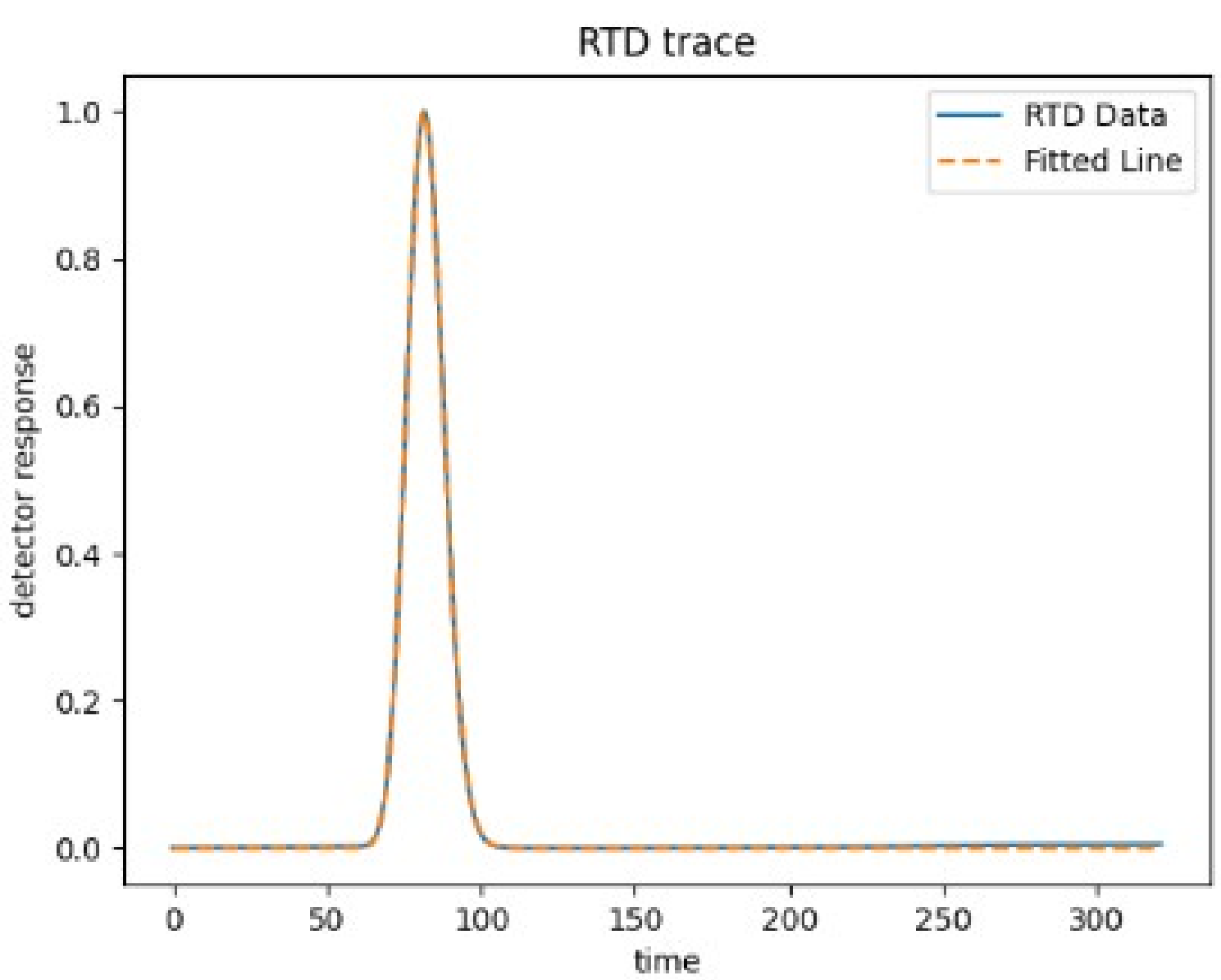

ESI-Figure 5: RTD Trace of a 10 mL 1.5mm ID reactor tube, of THF Flow rate 0.125 mL min^{-1} with a 100 µL toluene tracer.

Experimental Section

Methyl acrylate, 1,4-dioxane, 2,2'-azobis(isobutyronitrile) (AIBN), were purchased from Merck. The RAFT agent, 2-(((butylthio)carbonothioyl)thio)propanoic acid (PABTC) was synthesized according to previously reported literature.

GPC samples were recorded using a modified Agilent Infinity II instrument equipped with differential refractive index (DRI). The system was equipped with a PLgel Mixed D column (300 × 7.5 mm^2) and a PLgel 5 μm guard column. The eluent was THF. Samples were run at 1 mL/min at 30 °C. Poly(methyl methacrylate) standards (Agilent EasiVials) were used for calibration. Experimental molar mass (M_n, M_w, SEC) and dispersity (*Đ*) values of synthesized polymers were determined by conventional calibration using Agilent OpenLabs software.

Determination of Kinetics of RAFT Polymerization in batch with Online Monitoring

A flask was charged with DMA (9.2 g, 92.8 mmol), 1,4 –dioxane (75 g), 2-(butylthiocarbonothioylthio)propanoic acid (133 mg 0.56 mmol) and 4,4′-azobis (4-cyanovaleric acid) (15.7 mg, 0.062 mmol)(ACVA) before being de- oxygenated using nitrogen for 10 minutes. The flask was connected to a dual piston HPLC pump and the solution flowed through the 6-port, 2-position switching valve of an online GPC and returned to the flask, the reaction mixture was circulated through this loop at 1 mL min^{-1} for the duration of the experiment. The Reaction mixture was heated to 70 ˚C and stirred at 150 rpm for 24 hours. The reaction was sampled every 15 minutes by the injection of 100 uL of reaction mixture into the online GPC.

High throughput CCTP of MMA Reaction condition screening using two pumps.

To two flasks was added methyl methacrylate (100 g, 1.0 mol), 2,2'-azobis(isobutyronitrile (250 mg, 1.5 mmol) and toluene (100 g). To one of these flasks was also added CoBF (3.84 mg, 0.01 mmol), before both flasks were de-oxygenated with nitrogen for 30 minutes. Each flask was connected to an individual dual piston HPLC pump and flowed together at a combined flow rate of 0.5 mL min^{-1} for 10 hours, through a tubular reactor at 70˚C. The individual contribution of each pump was stepped from 90% to 0% for the solution containing no CoBF and 10% to 100% for the solution containing CoBF, in 10% increments every 60 minutes. The flow line was sampled with 100 uL of reaction mixture taken every 15 minutes and injected into the online GPC.

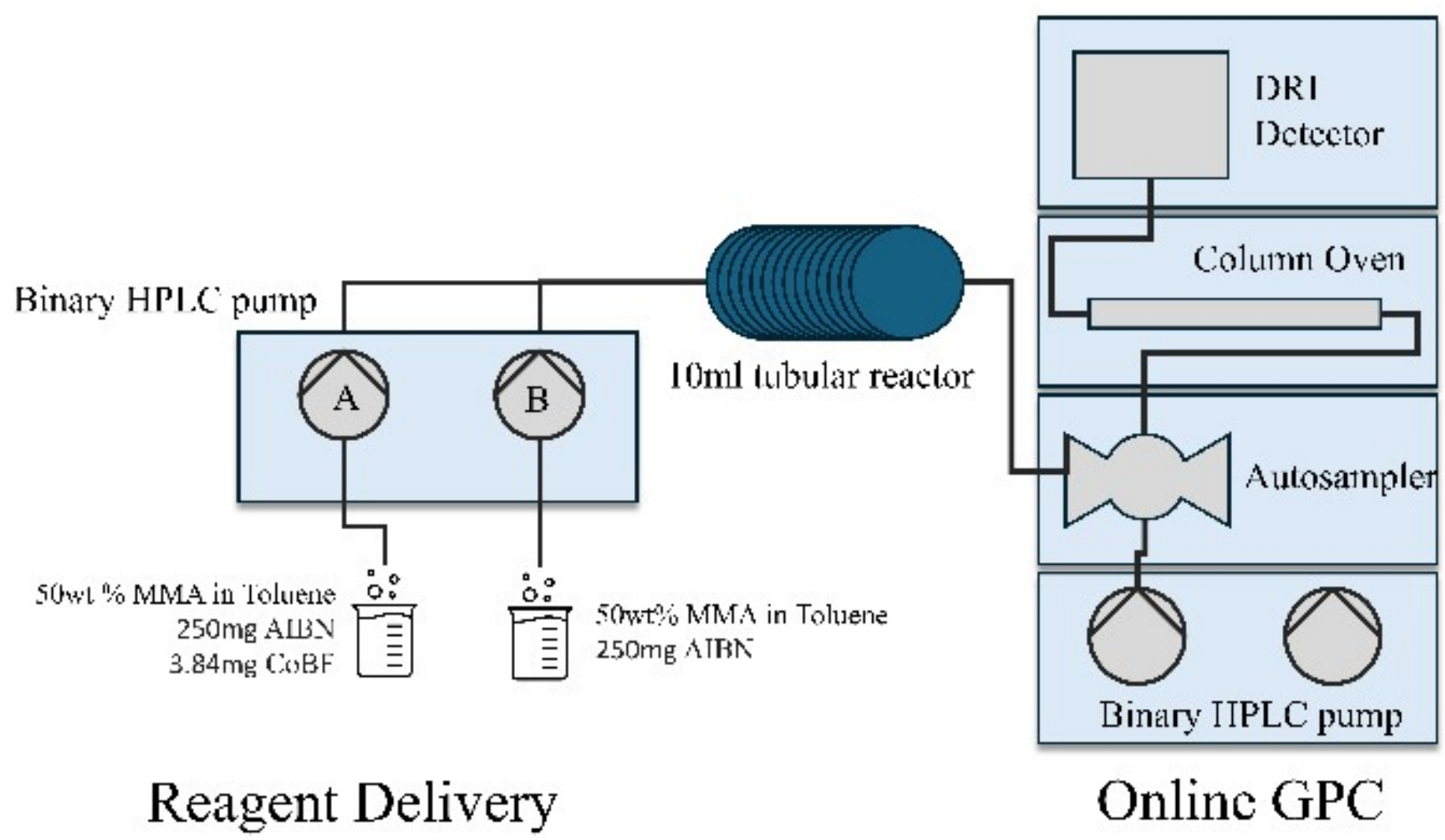

ESI-Figure 6. Schematic diagram of a two-pump reaction set up with online GPC used to determine the chain transfer constant of the CCTP of MMA in toluene

Table 1: RAW -Molecular weight data for the RAFT polymerization of MA moderated with PABTC and initiated with AIBN at 70˚C ranging from initiation to T= 20 hours at intervals of 15 minutes

Run	time	Mn	Mw	PDI	Area	Conc	Conv
number	(Mins)	g mol^{-1}	g mol^{-1}			mg mL^{-1}	%
0	15	877.4912	881.8684	1.004988	12.19458	0.005964	0.005421

1	30	991.1448	1012.317	1.021361	33.5178	0.016391	0.014901
2	45	2504.833	3015.869	1.20402	5665.966	2.770842	2.518947
3	60	3506.608	4213.62	1.201623	15328.9	7.496333	6.814848
4	75	4442.468	5279.337	1.188379	24204.46	11.83677	10.7607
5	90	5293.466	6222.98	1.175596	31742.59	15.52316	14.11197
6	105	6257.562	7288.628	1.164771	40245.64	19.68143	17.89221
7	120	7321.614	8394.167	1.146491	47520.03	23.23885	21.12622
8	135	8236.202	9374.89	1.138254	54679.5	26.74006	24.30915
9	150	9007.976	10193.13	1.131568	60880.74	29.77267	27.06606
10	165	9800.368	11028.16	1.12528	66669.1	32.60336	29.63942
11	180	10497.01	11777.93	1.122027	72649.46	35.52796	32.29814
12	195	11144.45	12501.11	1.121734	78606.65	38.44122	34.94656
13	210	11730.17	13165.16	1.122334	84109.45	41.13226	37.39297
14	225	12187.75	13686.49	1.122971	89043.61	43.54523	39.58657
15	240	12639.66	14214.39	1.124586	93925.87	45.93281	41.7571
16	255	12970.98	14609.37	1.126312	98499.02	48.16923	43.79021
17	270	13315.75	15035.13	1.129123	102902.2	50.32251	45.74774
18	285	13756.24	15579.7	1.132555	107339.6	52.49256	47.72051
19	300	13943.58	15839.3	1.135957	111332.7	54.4453	49.49573
20	315	14152.64	16116.98	1.138797	115003.7	56.24055	51.12777
21	330	14493.73	16534.77	1.140822	118068	57.7391	52.49009
22	345	14570.33	16685.98	1.145203	122273.4	59.7957	54.35973
23	360	14990.13	17208.4	1.147982	125082.3	61.16932	55.60847
24	375	14957.54	17200.79	1.149975	127457.2	62.33073	56.6643
25	390	15250.87	17572.53	1.152231	129789.9	63.47151	57.70138
26	405	15370.49	17766.75	1.1559	132735.6	64.91205	59.01095
27	420	15395.32	17815.06	1.157174	134544.3	65.79657	59.81506
28	435	15664.13	18167.42	1.15981	136814.4	66.90672	60.8243
29	450	15596.39	18121.64	1.161913	138826	67.89042	61.71857
30	465	15812.47	18402.96	1.163826	140884.1	68.89693	62.63357
31	480	15870.8	18517.14	1.166742	142986.7	69.92516	63.56832
32	495	15931.97	18601.6	1.167565	144312	70.57328	64.15753
33	510	16061.59	18806.21	1.170881	146760.7	71.77075	65.24614
34	525	16224.34	19016.58	1.172102	148052.8	72.40263	65.82057
35	540	16132.14	18947	1.174488	149796.1	73.25516	66.5956
36	555	16255.79	19113.27	1.175782	151021	73.85419	67.14018
37	570	16335.79	19220.05	1.176561	151948.1	74.30757	67.55234
38	585	16331.61	19259.54	1.17928	153853.2	75.23922	68.39929
39	600	16252.22	19203.34	1.181582	155319.4	75.95628	69.05116

40	615	16509.03	19541.46	1.183683	156690.8	76.62689	69.66081
41	630	16453.74	19498.87	1.185072	157801.6	77.17015	70.15468
42	645	16739.82	19868.05	1.186873	159089.5	77.79996	70.72724
43	660	16693.75	19837.75	1.188334	160117.4	78.30265	71.18423
44	675	16670.95	19835.63	1.189832	161131.7	78.79867	71.63515
45	690	16884.03	20102.36	1.190614	161840.7	79.14537	71.95033
46	705	16898.31	20128.95	1.191182	162472	79.4541	72.231
47	720	16884.72	20133.56	1.192413	163012	79.71818	72.47108
48	735	16879.6	20165.25	1.194652	164456.8	80.42473	73.11339
49	750	17025.8	20364.63	1.196104	165344.1	80.85866	73.50787
50	765	16977.15	20300.45	1.195752	165350.9	80.862	73.51091
51	780	17168.03	20560.26	1.19759	166478.1	81.41322	74.01202
52	795	17033.58	20405.67	1.197967	166902.3	81.62068	74.20062
53	810	17146.61	20577.94	1.200117	167934.9	82.12565	74.65969
54	825	17155.83	20589.78	1.200163	168109.7	82.21113	74.73739
55	840	17108.82	20556.49	1.201514	168850.2	82.57323	75.06658
56	855	17166.26	20651.53	1.20303	169659.6	82.9691	75.42646
57	870	17192.49	20701.39	1.204095	170211.6	83.23904	75.67186
58	885	17183.52	20692.61	1.204212	170377.6	83.32022	75.74566
59	900	17291.42	20843.35	1.205416	171106.1	83.67648	76.06953
60	915	17359.36	20930.06	1.205693	171277	83.76002	76.14548
61	930	17473.08	21079.24	1.206384	171712.3	83.97289	76.33899
62	945	17479.81	21106.1	1.207456	172252	84.23683	76.57894
63	960	17411.87	21043.32	1.208562	172799.9	84.5048	76.82255
64	975	17444.69	21090.62	1.209	172955.7	84.58096	76.89178
65	990	17449.26	21102.75	1.209378	173093.2	84.6482	76.95291
66	1005	17530.82	21226.46	1.210809	173852.5	85.01955	77.2905
67	1020	17507.49	21205.59	1.21123	174057.5	85.11982	77.38165
68	1035	17404.17	21081.54	1.211292	173977.7	85.08079	77.34618
69	1050	17375.42	21057.42	1.211908	174240	85.20904	77.46277
70	1065	17621.67	21342.78	1.211166	174126	85.15329	77.41208
71	1080	17592.73	21328.08	1.212324	174530.8	85.35124	77.59204
72	1095	17524.73	21264.23	1.213384	174983.6	85.57266	77.79333
73	1110	17476.08	21215.43	1.21397	175225.3	85.69089	77.90081
74	1125	17636.92	21431.77	1.215165	175584.1	85.86633	78.0603
75	1140	17458.2	21206.4	1.214696	175389.3	85.7711	77.97373
76	1155	17539.8	21332.08	1.21621	175937.3	86.03909	78.21736
77	1170	17647.29	21452.82	1.215644	175600.2	85.87421	78.06746
78	1185	17628.4	21435.61	1.215971	175760.4	85.95259	78.13872

79	1200	17526.5	21346.31	1.217945	176418.9	86.2746	78.43145
80	1215	17629.51	21490.47	1.219006	176810.6	86.46614	78.60558
81	1230	17377.75	21202.25	1.220081	177245.5	86.67881	78.79892
82	1245	17431.62	21255.99	1.219393	176892.8	86.50637	78.64215
83	1260	17344.05	21137.94	1.218744	176441.3	86.28554	78.4414
84	1275	17575.65	21447.17	1.220278	176963.4	86.54089	78.67354
85	1290	17496.12	21338.95	1.219639	176476	86.3025	78.45682
86	1305	17368.07	21217.86	1.22166	177315.9	86.71324	78.83022
87	1320	17322.16	21142.31	1.220535	176598	86.36218	78.51108
88	1335	17443.86	21302.05	1.221178	176824.9	86.47316	78.61196
89	1350	17255.55	21074.94	1.221343	176749.6	86.4363	78.57845
90	1365	17268.81	21089.28	1.221236	176765.6	86.44413	78.58557
91	1380	17495.28	21398.79	1.223118	177499.5	86.80303	78.91184
92	1395	17292.89	21127.63	1.221753	176659.5	86.39227	78.53843
93	1410	17337.79	21205.79	1.223097	177099.8	86.6076	78.73418
94	1425	17252.45	21132.94	1.224924	177574.1	86.83951	78.94501
95	1440	17230.29	21074.51	1.223108	176715.5	86.41965	78.56332
96	1455	17158.71	20993.82	1.223508	176839.2	86.48014	78.61831
97	1470	17207.38	21055.57	1.223636	176723.3	86.42344	78.56676
98	1485	17211.72	21060.39	1.223608	176669	86.39693	78.54266
99	1500	17260.37	21143.18	1.224955	177049.2	86.58281	78.71165

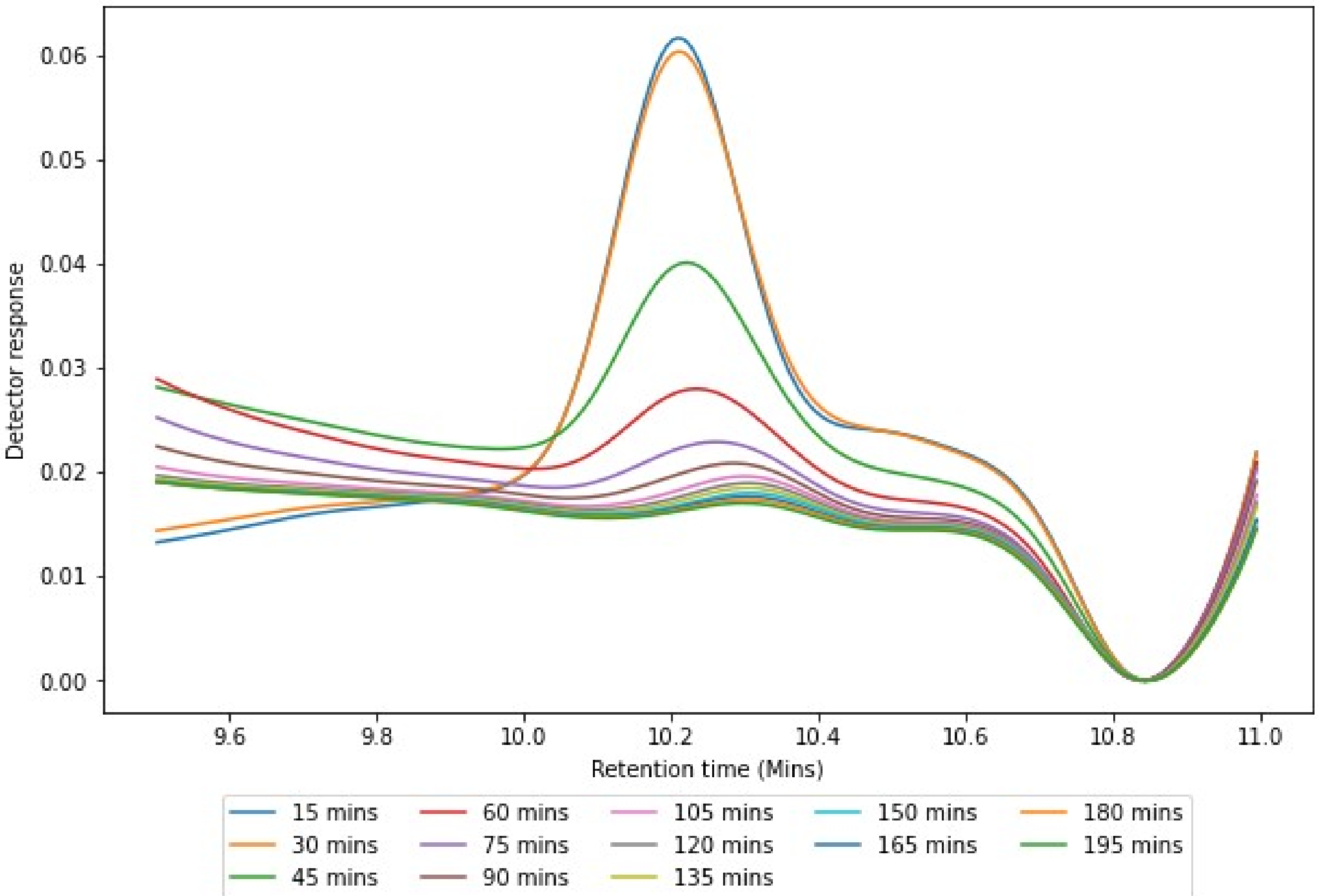

ESI-Figure 7 Overlayed normalized GPC traces from RAFT polymerization of MA moderated with PABTC and initiated with AIBN at 70˚C ranging from initiation to T= 20 hours at intervals of 15 minutes, closer view of the peak attributed to the RAFT Agent.

Table 2: Group A CCTP 250 mg AIBN, τ_r = 20 mins 70 ˚C

[CoBF]/[MMA](ppm)	$M_{n,GPC}$ (g/mol)	$M_{w,GPC}$ (g/mol)	Đ
10	260	455	1.75
9	310	574	1.88
8	300	570	1.88
7	330	620	1.92
6	360	700	1.95
5	420	860	2.06
4	500	1070	2.13
3	640	1440	2.26
2	890	2160	2.42
1	1500	4120	2.75

Table 3 :Group B CCTP 250 mg AIBN, τ_r= 40 mins 70 ˚C

[CoBF]/[MMA](ppm)	$M_{n,GPC}$ (g/mol)	$M_{w,GPC}$ (g/mol)	Đ
10	290	510	1.77
9	340	640	1.90
8	530	1220	2.29
7	440	910	2.06
6	480	1030	2.17
5	570	1270	2.24
4	730	1590	2.18
3	1040	2140	2.05
2	1490	2950	1.99
1	2380	4800	2.02

Table 4 Group C cctp 250 mg AIBN, τ_r = 20 mins 80 ˚C

[CoBF]/[MMA](ppm)	$M_{n,GPC}$ (g/mol)	$M_{w,GPC}$ (g/mol)	Đ
10	610	980	1.60
9	650	1080	1.65
8	720	1220	1.70
7	770	1340	1.73
6	860	1500	1.75
5	1020	1800	1.77
4	1260	2250	1.78
3	1540	2760	1.79
2	1720	3160	1.84
1	3270	5780	1.77

Table 5 Group D CCTP 250 mg AIBN, τ_r = 20 mins 70 ˚C

[CoBF]/[MMA](ppm)	$M_{n,GPC}$ (g/mol)	$M_{w,GPC}$ (g/mol)	Đ
10	950	1270	1.33
9	1030	1450	1.41
8	1130	1690	1.48
7	1200	1830	1.52
6	1320	2070	1.57
5	1510	2400	1.59
4	1790	2790	1.56
3	2130	3340	1.57
2	3670	6150	1.68
1	6520	10020	1.54

Table 6 group A 70 ˚C, Tr = 60 mins ACVA 47 mg

[PABTC]/[DMA]	$M_{n,GPC}$ (g/mol)	$M_{w,GPC}$ (g/mol)	Đ
0.0120	1900	2200	1.17
0.0106	2300	2700	1.17
0.0093	2800	3200	1.15
0.0080	3000	3500	1.16
0.0067	4000	4800	1.19
0.0053	4900	5800	1.20
0.0040	6700	8200	1.23
0.0027	8400	14000	1.36
0.0013	10500	14000	1.36

Table7 group B 70 ˚C, Tr = 30 mins ACVA 47 mg

[PABTC]/[DMA]	$M_{n,GPC}$ (g/mol)	$M_{w,GPC}$ (g/mol)	Đ
0.0120	820	970	1.18
0.0106	970	1140	1.18
0.0093	1350	1600	1.18
0.0080	1660	2000	1.20
0.0067	2370	2780	1.17
0.0053	2840	3400	1.20
0.0040	3020	3640	1.20
0.0027	4510	5780	1.28
0.0013	7110	9660	1.36

Table 8 Group C 70 ˚C, Tr = 60 mins ACVA 30 mg

[PABTC]/[DMA]	$M_{n,GPC}$ (g/mol)	$M_{w,GPC}$ (g/mol)	Đ
0.0120	2380	3240	1.36
0.0106	2510	2990	1.19
0.0093	2700	3150	1.16
0.0080	3190	3720	1.16
0.0067	4010	4760	1.19
0.0053	4640	5600	1.21
0.0040	6440	8060	1.25
0.0027	9860	12450	1.26
0.0013	14400	19280	1.34

Table 9 Group D 80 ˚C, Tr = 60 mins, ACVA 30 mg

[PABTC]/[DMA]	$M_{n,GPC}$ (g/mol)	$M_{w,GPC}$ (g/mol)	Đ
0.0120	2680	3320	1.24
0.0106	3190	3570	1.12
0.0093	3400	3850	1.13
0.0080	3820	4330	1.13
0.0067	4330	4940	1.14
0.0053	5230	6130	1.17
0.0040	7190	8810	1.23
0.0027	12020	15080	1.26

0.0013	16510	22130	1.34

Python script for non-linear least squares determination of Peclet number and Average residence time

This script calls a single CSV which must contain Time data in the first column and detector response in the second. There should be no data headers, and this code will skip the first row to remove x = 0.

```
def run_calc():  #function to be called

    RTD_data = pd.read_csv(r'YOUR DATA LOCATION', skiprows=1, header=None) #import data from CSV
    x_vals = RTD_data.iloc[:,0].values #exctract X vals from column 1
    y_vals = RTD_data.iloc[:,1].values #exctract Y vals from column 2
    y_vals = (y_vals - np.min(y_vals)) / (np.max(y_vals) - np.min(y_vals)) #update Y vals with normalized Y values

    optimised_perams = RTD_CALC(x_vals,y_vals)  #called RTD function, defined below to obtain Tr and Pe
    plot_data(x_vals, y_vals, optimised_perams) #plotting original data and calculated line
    print ("optimised_perams") #prints calculated parameters to console

def RTD_CALC(x_vals,y_vals):

    def peclet_calc(params, x_vals, y_vals):
        Tr, Pe = params #defines parameters to optimise
        calc_y = (np.sqrt(Pe / (4 * np.pi * (x_vals / Tr)))) * np.exp(-(((1 - (x_vals/ Tr))**2) / ((4 * (x_vals / Tr)) / Pe))) # equation for axial diffusion
        norm_calc_y_vals = (calc_y - np.min(calc_y)) / (np.max(calc_y) - np.min(calc_y)) #normalising calculated Y values
        dif = y_vals - norm_calc_y_vals #determining difference
        return dif

    init_t = get_x_of_max_y(x_vals,y_vals) #locates highest value of Y data and returns the corresponding value for X
    initial_guess = [init_t, 4543] # creates initial set of parameters where 1st entry is Tr and 2nd is Pe, initial value for Pe was chosen at random
    result = least_squares(peclet_calc, initial_guess, args=(x_vals, y_vals)) # performs least squares function

    optimized_params = result.x #exctacts parameters
    print("Optimized parameters:", optimized_params)  #prints parameters to console
    return optimized_params

def get_x_of_max_y(x_vals,y_vals): #function for locating highest value of Y data and returns the corresponding value for X
    idx_max_y = np.argmax(y_vals)
    return x_vals[idx_max_y]


def plot_data(x_vals, y_vals, optimized_params): #function for plotting data
    plt.plot(x_vals, y_vals, label='RTD Data')

    # Calculate the fitted line using the optimized parameters
    Tr, Pe = optimized_params
    print (Tr)
    print (Pe)
    fitted_y_vals = (np.sqrt(Pe / (4 * np.pi * (x_vals / Tr)))) * np.exp(-(((1 - (x_vals/ Tr))**2) / ((4 * (x_vals / Tr)) / Pe)))
    fitted_y_vals = (fitted_y_vals - np.min(fitted_y_vals)) / (np.max(fitted_y_vals) - np.min(fitted_y_vals))

    # Plot the fitted line
    plt.plot(x_vals, fitted_y_vals, label='Fitted Line', linestyle='--')

    plt.xlabel('time')
    plt.ylabel('detector response')
    plt.title('RTD trace')
    plt.legend()
    plt.show()
```

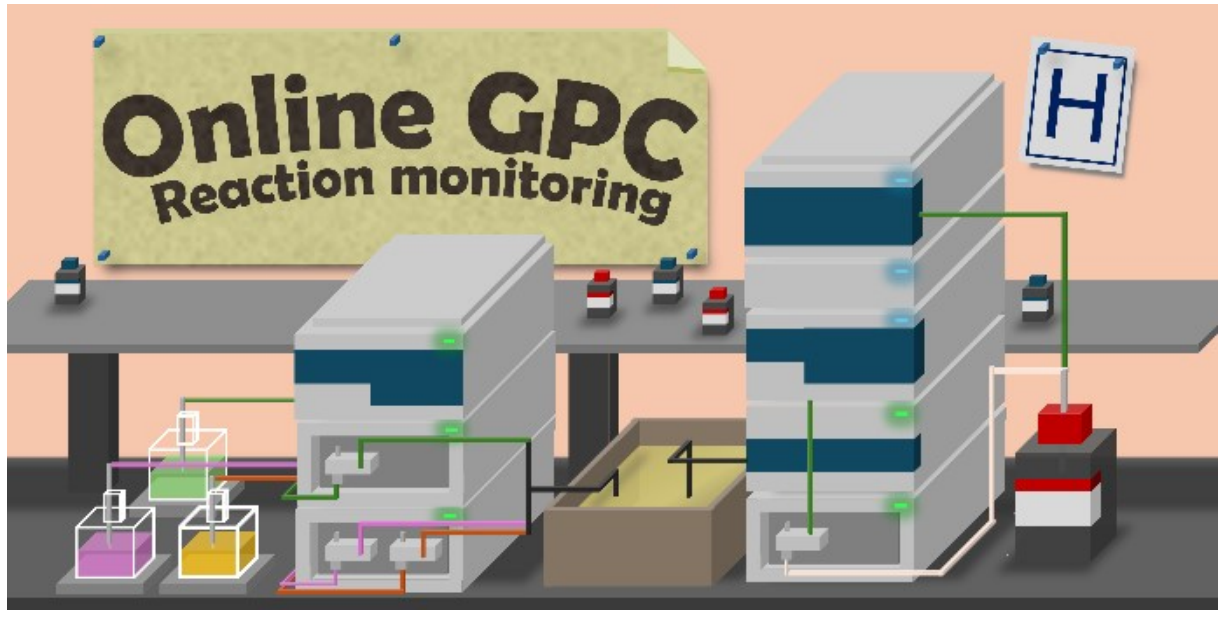
Online GPC
Reaction monitoring
H
